# Evidence of Guanidines Potential against *Leishmania (Viannia) braziliensis*: Exploring In Vitro Effectiveness, Toxicities and of Innate Immunity Response Effects

**DOI:** 10.3390/biom14010026

**Published:** 2023-12-24

**Authors:** Luana Ribeiro dos Anjos, Vanessa Maria Rodrigues de Souza, Yasmim Alves Aires Machado, Vitor Moreira Partite, Mohammed Aufy, Geovane Dias Lopes, Christian Studenik, Carlos Roberto Alves, Gert Lubec, Eduardo Rene Perez Gonzalez, Klinger Antonio da Franca Rodrigues

**Affiliations:** 1 Fine Organic Chemistry Lab, School of Sciences and Technology, São Paulo State University (UNESP), Presidente Prudente 19060-080, Brazil; luana.anjos@unesp.br (L.R.d.A.); vitor.partite@unesp.br (V.M.P.); 2 Infectious Disease Laboratory—LADIC, Federal University of Parnaíba Delta—UFDPar, Campus Ministro Reis Velloso, São Benedito, Parnaíba 64202-020, Brazil; rodriguesvanessa745@ufpi.edu.br (V.M.R.d.S.); machado03@ufpi.edu.br (Y.A.A.M.); 3 Department of Pharmaceutical Sciences, Division of Pharmacology and Toxicology, University of Vienna, Josef Holaubek Platz 2, UZAII (2D 259), 1090 Vienna, Austria; mohammed.aufy@univie.ac.at (M.A.); christian.studenik@univie.ac.at (C.S.); 4 Laboratório de Biologia Molecular e Doenças Endêmicas, Fundação Oswaldo Cruz, Instituto Oswaldo Cruz, 4365, Manguinhos, Rio de Janeiro 21040-900, Brazil; geovane.dl@gmail.com (G.D.L.); klvess@gmail.com (C.R.A.); 5 Department of Neuroproteomics, Paracelsus Medical University, 5020 Salzburg, Austria; gert.lubec@lubeclab.com

**Keywords:** guanidine derivatives, *Leishmania (Viannia) braziliensis*, immunomodulation, organ and cell toxicity

## Abstract

Leishmaniasis is a complex group of infectious and parasitic diseases that afflict many thousands of individuals across five continents. Leishmaniasis treatment remains a challenge because it relies on drugsknown for their high toxicity and limited efficacy, making itimperative to identify new molecules that offer greater effectiveness and safety. This study sought to explore the impact of seven synthetic guanidine derivatives (LQOF-G1, LQOF-G2, LQOF-G6, LQOF-G7, LQOF-G32, LQOF-G35 and LQOF-G36) onthe parasite *Leishmania (Viannia) braziliensis* and in vitro macrophage infection by this parasite, as well as cytotoxic approaches in vitro models of mammalian host cells and tissues. The synthesized compounds showed purity ≥ 99.65% and effectively inhibited parasite growth. LQOF-G1 proved the most potent, yielding the best half-maximal inhibitory concentration (IC_50_) values against promastigotes (4.62 μmol/L), axenic amastigotes (4.27 μmol/L), and intracellular amastigotes (3.65 μmol/L). Notably, the antileishmanial activity of LQOF-G1, LQOF-G2, and LQOF-G6 was related to immunomodulatory effects, evidenced by alterations in TNF-α, IL-12, IL-10, nitric oxide (NO), and reactive oxygen species (ROS) levels in the supernatant of culture macrophages infected with *L. (V.) braziliensis* and coincubated with these compounds. LQOF-G2 and LQOF-G36 compounds exhibited vasodilator and spasmolytic effects at higher concentrations (≥100 μmol/L). Generally, LQOF-G1, LQOF-G2, and LQOF-G32 compounds were found to be nontoxic to assessed organs and cells. No toxic effects were observed in human cell lines, such as HEK-293, CaCo-2 and A549, at concentrations ≥ 500 μmol/L. Collectively, data have shown unequivocal evidence of the effectiveness of these compounds against *L. (V.) braziliensis* parasite, one of the causative agents of Tegumentary Leishmaniasis and Mucocutaneous Leishmaniasis in America.

## 1. Introduction

Leishmaniasis is a group of infectious and parasitic diseases caused by protozoan of the genus *Leishmania*. They are transmitted through the blood meal of insect vectors of the genus *Phlebotomus* (in Europe, Asia, and Africa) and *Lutzomyia* (in the Americas) [1]. According to the World Health Organization (WHO), in 2022, leishmaniasis is endemic in 98 countries, affecting more than 12 million people and resulting in an estimated 1.5 to 2 million new cases annually. These diseases are linked to factors such as poor housing, limited financial resources, population displacement, malnutrition, and weakened immune systems, which categorizes them as neglected tropical diseases (NTDs) [2].

More than 20 *Leishmania* spp. are known human pathogens, and they give rise to diverse clinical manifestations that depend on both the specific species involved and the host’s immune system [3]. Cutaneous Leishmaniasis (CL) typically manifests as papular/nodular and/or ulcerative skin lesions. Mucocutaneous leishmaniasis (ML) is characterized by the destructive inflammation of mucous membranes. Visceral Leishmaniasis (VL) is considered the most lethal clinical form and results in systemic manifestations affecting the liver, spleen, hematogenous, and lymphatic systems. Post-kala-azar dermal leishmaniasis (PKDL) is a complication of visceral leishmaniasis (VL); it is characterized by a macular, maculopapular, and nodular rash in a patient who has recovered from VL [3]. Among the *Leishmania* species of medical significance, *Leishmania* (*Viannia*) *braziliensis* holds particular importance. This parasite is related to American Tegumentary Leishmaniasis (ATL), which encompasses both CL and ML clinical presentations. This species is prevalent throughout Latin America and is particularly widespread in Brazil [4].

Despite their clinical and epidemiological significance, these diseases pose a considerable challenge to public health due to the limited and outdated treatment options currently at hand. The primary medications used include pentavalent antimonials (Sb+5), which are available in two formulations: meglumine antimoniate and sodium stibogluconate. These drugs are administered parenterally, either intramuscularly or intravenously, and have been in use since the 1940s for all forms of leishmaniasis [5]. However, they are associated with a range of side effects, including musculoskeletal pain, headache, nausea, cardiotoxicity, hepatotoxicity, nephrotoxicity, and pancreatitis [6,7,8].

Miltefosine, known as hexadecylphosphocholine, is another first-line drug and represents an oral treatment option approved for both cutaneous and visceral leishmaniasis [9]. However, it also has its drawbacks, causing some unwanted effects [10]. Second-line drugs, such as Amphotericin B and Pentamidine, are available but come with their own disadvantages, including increased toxicity compared to antimonials [11]. Another drug option is Paromomycin, an aminoglycoside antibiotic with leishmanicidal properties, but it is also associated with considerable side effects [11].

**Figure 1:**
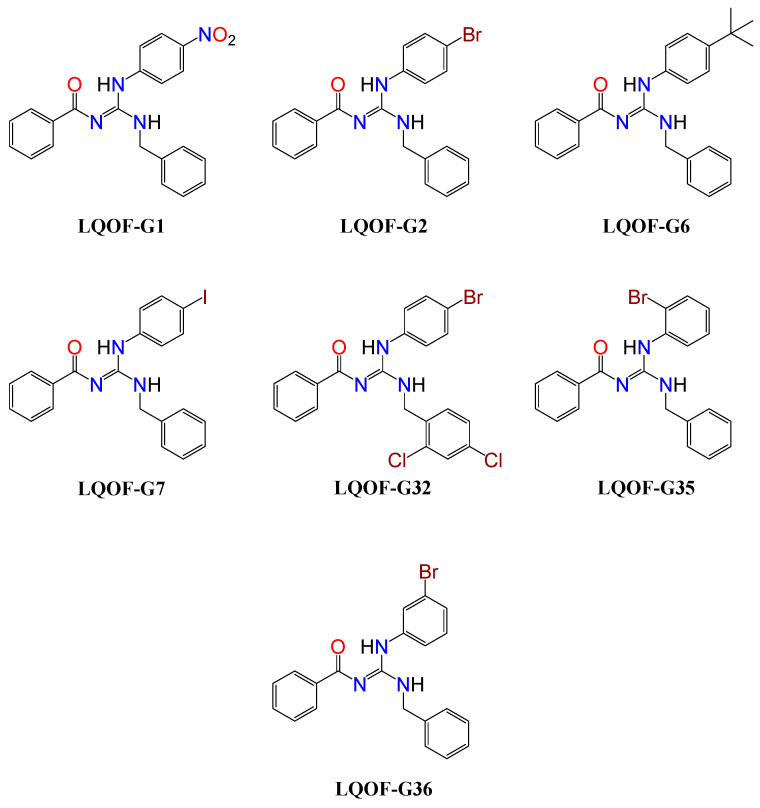
Structure of the seven guanidines compounds LQOF-G1, LQOF-G2, LQOF-G6, LQOF-G7, LQOF-G32, LQOF-G35 and LQOF-G36.

Therefore, new drug proposals for leishmaniasis treatment are necessary. Previous results show the potential of several guanidine-derived compounds, such as the (Z)-N-benzoyl-N-benzyl-N-(4-bromophenyl)guanidine LQOF-G2 (Figure 1), are promising agents against cutaneous leishmaniasis, with a good selectivity index (SI = 131.8) [12]. In vivo testing in binomial BALB/c-*Leishmania (Leishmania) amazonensis* treated with this compound resulted in a remarkable 46% reduction in the parasite load at 0.13 mg/kg/day [13]. Furthermore, studies have indicated that guanidines can act as inhibitors of enzymes crucial for the virulence of leishmaniasis, as shown by (Z)-N-benzoyl-N-benzyl-N-(4-tert-butylphenyl)guanidine (LQOF-G6, Figure 1), which demonstrated 73% inhibition of the enzyme LmCPB2.8ΔCTE.

Building upon these previous findings, this study advances with new findings of the biological effects of guanidine compounds LQOF-G1, LQOF-G2, LQOF-G6, LQOF-G7, LQOF-G32, LQOF-G35 and LQOF-G36 (Figure 1). This study investigates the in vitro effect of these compounds against *L. (V.) braziliensis*, as well as cytotoxicity assays in mammalian host cells and tissues and innate immunity response effects. Furthermore, the present work presents the synthesis and structural characterization of a new guanidine compound, LQOF-G36. Data obtained in this study with *L. (V.) braziliensis* contribute to proving the spectrum of activity of this series of guanidine compounds against *Leishmania* spp.

## 2. Results and Discussion

The focus of this study was to explore and compare guanidine compound potentiality as a leishmanicidal agent since most of the presented compounds are already published, with results of antileishmania activity against *L. (L.) amazonensis* [12], *L. (L.) major* [13] and *L. (L.) infantum* [14]. In fact, compounds incorporating guanidine moieties are biologically effective with a wide array of applications and with pharmacological importance in drug activity [15]. This study presents new data on guanidine compounds efficacies against parasite belonging to the *Viannia* subgenus, the *L. (V.) braziliensis*. Together with previous data on guanidine efficacy against parasites from *Leishmania* subgenus, *L. (L.) amazonensis* [12] and *L. (L.) infantum* [14], the spectrum of activity of guanidine compounds against the *Leishmania* spp. subgenera involved in the etiology of CL and VL is investigated here.

### 2.1. Synthesis and Structural Characterization of LQOF-G35 and LQOF-G36

**Scheme 1:**
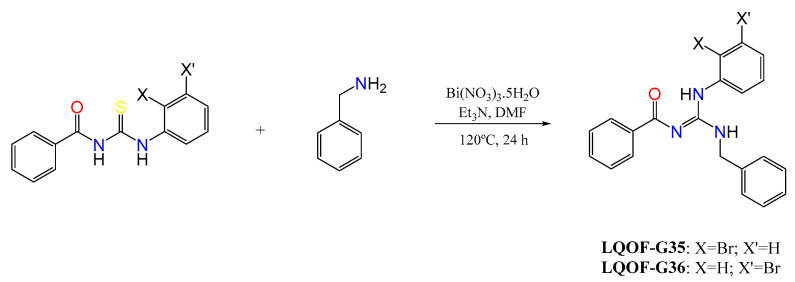
Synthesis of the compounds LQOF-G35 and LQOF-G36.

The synthesis of guanidines LQOF-G1, LQOF-G2, LQOF-G6, LQOF-G7 and LQOF-G32 have been reported earlier [12,13,14]. Considering the viability of this method and the high reaction yields, the synthesis of new guanidine compounds, LQOF-G35 and LQOF-G36 (Scheme 1), was carried out according to previously described methods [12].

The synthesis of this new compound was due to the good results reported for LQOF-G2, a brominated guanidine. Therefore, aiming at a potential antileishmanial and based on the promising results of brominated compounds, compounds LQOF-G35 and LQOFG-36 were prepared to explore the effect of changing the position of the 4-Br atom in LQOF-G32 to the ortho and meta position.

Compounds LQOF-G35 and LQOF-G36 underwent a thorough characterization process, which involved analysis of LC-UV/MS (Figures S7 and S8), ESI(+)-MS (Figures S9 and S11), ESI(+)-MS/MS (Figures S10 and S12), and NMR data (Tables S1 and S2). The structure of these compounds was elucidated with absolute certainty. Detailed information regarding the complete structural characterization can be found in the Supplementary Materials.

### 2.2. In Vitro Antileishmanial Efficacy of Guanidines and Their Cytotoxicity

In a preliminary experiment, we investigated seven guanidine compounds for their effectiveness against the promastigote forms of *L*. (*V*.) *braziliensis*, while also assessing cytotoxicity against RAW 264.7 macrophages. The study aimed to identify the most active and selective compounds against the parasite.

All seven guanidines exhibited antileishmanial activity against promastigotes, with IC_50_ values ranging from 4.6 μmol/L (LQOF-G1) to 25.9 μmol/L (LQOF-G35). The compounds displaying the lowest IC_50_ values and highest selectivity index were chosen for testing in axenic amastigotes. These compounds also demonstrated activity against axenic amastigotes, with EC_50_ values ranging from 4.3 µmol/L (LQOF-G1) to 6.1 µmol/L (LQOF-G7). For a comprehensive overview of the IC_50_, EC_50_, and CC_50_ values, refer to Table 1, which also includes meglumine antimoniate and amphotericin B as reference drugs for comparison.

In previous works, the antipromastigote activity against *L. (L.) amazonensis* was evaluated for the compounds LQOF-G1 (IC_50_ 11.5 µmol/L), LQOF-G2 (19.6 µmol/L), LQOF-G6 (25.9 µmol/L) and LQOF-7 (15.9 µmol/L) [12], and on *L. (L.) infantum* for the compounds LQOF-G2 (12.7 µmol/L), LQOF-G6 (24.4 µmol/L) and LQOF-7 (23.6 µmol/L) [14]. The difference in the sensitivity of species to guanidine derivatives may be related to biochemical/molecular differences between the species studied, which present different levels of intrinsic resistance acquired during passage through hosts. This difference is observed in most studies testing new drugs and sensitivity to reference drugs [16].

Higher selectivity index values were observed for compounds containing specific chemical groups—nitro (LQOF-G1), *tert*-butyl (LQOF-G6), and bromine (LQOF-G2)—positioned in the ‘para’ position of the aniline moiety. To put this into context, when compared to reference drugs, LQOF-G1 exhibited remarkably 21.56-fold higher safety margin in relation to macrophages than meglumine antimoniate. These selectivity index values provide a robust representation of the in vitro efficacy of guanidines relative to the reference drugs, which demonstrated that both meglumine antimoniate and amphotericin B, despite being used in therapy, do not present a good margin of safety SI ≤ 2.0.

The compounds that displayed the lowest IC50 values against promatigotes of *L. (V.) braziliensis* and higher selectivity indexes (i.e., LQOF-G1, LQOF-G2, LQOF-G6, and LQOF-G7) were selected for further in-depth investigation and study with intramacrophage amastigotes. This was carried outin comparison with the values obtained for the molecules LQOF-G32, LQOF-G35 and LQOF-G36, which had lower SI values.

### 2.3. In Vitro Efficacy of Guanidines LQOF-G1, LQOF-G2, LQOF-G6 and LQOF-G7 against Intramacrophage Amastigotes

Once the effective and selective antileishmanial activity of guanidine derivatives on axenic forms of *L. (V.) braziliensis* was observed, the selected compounds (LQOF-G1, LQOF-G2, LQOF-G6 and LQOF-G7) were investigated using an in vitro model of macrophage infection. Antileishmanial activity modeling involving intramacrophage amastigotes is considered the most effective model for correlating in vivo effects since it closely mimics the infection of the primary cell type parasitized by *Leishmania* spp. [17]. The results of treatment with the guanidine derivatives LQOF-G1, LQOF-G2, LQOF-G6 and LQOF-G7 are shown in Table 1 and more detailed information can be found in the Supplementary Materials (Figure S13).

All the guanidine derivatives reduced the parasite survival rate in a concentration-dependent manner after 72 h of treatment, resulting in EC_50_ values of 3.65, 4.21, 3.89, and 8.25 μmol/L for the compounds LQOF-G1, LQOF-G2, LQOF-G6, and LQOF-G7, respectively (Table 1). Meglumine antimoniate and amphotericin B, which are first and second-line drugs in the treatment of leishmaniasis, presented respective EC_50_ values of 451.55 and 0.35 μmol/L. Despite its good activity, amphotericin B presented the lowest SI values, indicating significant toxicity to the host cell (CC_50_ = 0.55 μmol/L), which aligns with its observed adverse effects in clinical investigations [18].

### 2.4. Compounds LQOF-G1, LQOF-G2, and LQOF-G6 Induce a Host-Protective Cytokine Response and Stimulate an Increase in NO and ROS Levels

Given the reduction in the rate of macrophage infection promoted by treatment with the guanidine derivatives LQOF-G1, LQOF-G2, and LQOF-G6, we investigated whether their antiamastigote activity is associated with immune response modulation.

These guanidines were slightly more effective against intramacrophagic amastigotes than against axenic amastigotes. This difference may be indicative of macrophage activation mechanisms and microbicidal effects responsible for parasite death [17].

**Figure 2:**
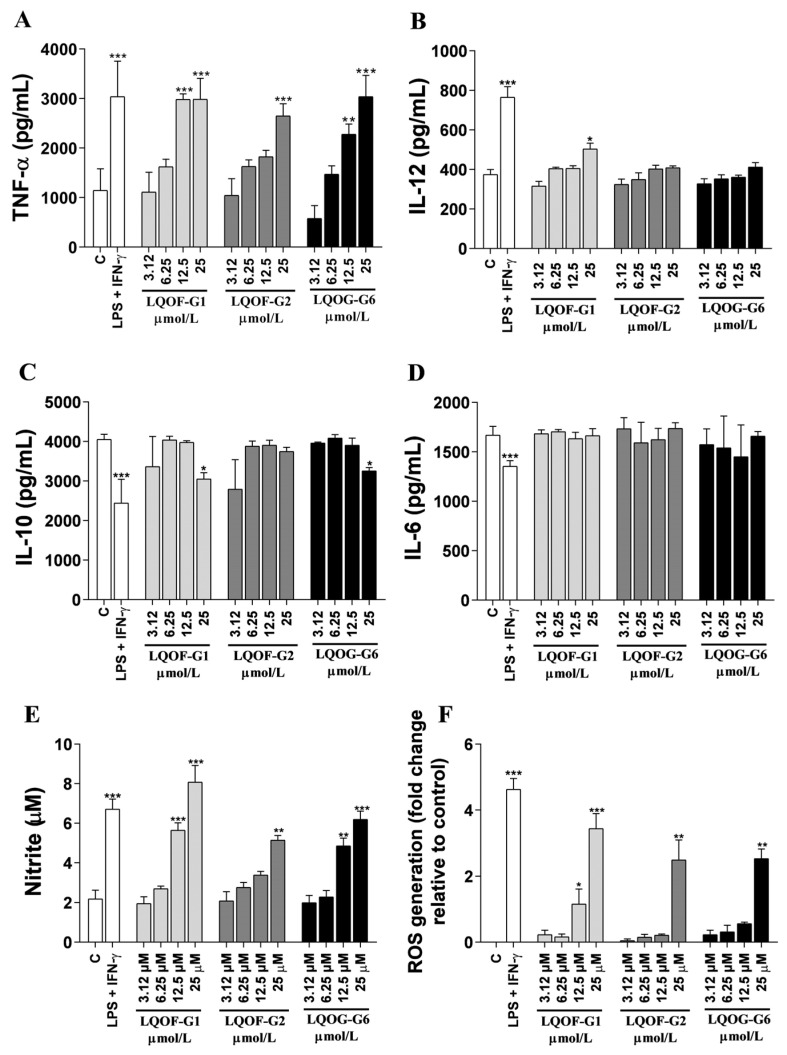
Immunomodulatory profile of LQOF-G1, LQOF-G2, LQOF-G6 in macrophages infected with *Leishmania* (*Viannia*) *braziliensis.* (**A**) TNF-α; (**B**) IL-12; (**C**) IL-10; (**D**) IL-6; (**E**) NO, and ROS (**F**) were evaluated using supernatant from cultured RAW 264.7 macrophages infected with *L.* (*V.*) *braziliensis*. Data represent the mean ± standard error of the mean of at least three independent experiments. Comparison between groups was performed by One-wayANOVA followed by Tukey’s post-test, where * *p* < 0.05; ** *p* < 0.01 and *** *p* < 0.001 compared to the control. LPS—lipopolysaccharide.

Macrophages are essential to the antileishmania immune response and play an important role in the process of susceptibility or resistance to leishmaniasis. The main lines of defense include both structural (increased phagocytosis and parasitophorous vacuole) and cellular (changes in cytokine, ROS, and NO levels) adaptations [17]. Proinflammatory cytokines, such as IL-1β, IFN-γ, TNF-α and IL-12 (Th1 response), are commonly related to resistance to infection since they induce an increase in microbicidal substances, such as ROS and NO. However, anti-inflammatory cytokines, such as IL-4, IL-6, and IL-10 (Th2 response), inhibit the production of proinflammatory cytokines and are linked to susceptibility to *Leishmania* spp. infections [19,20]. The guanidine derivatives promoted a protective host response, increased proinflammatory cytokines in the Th1 response, such as TNF-α (LQOF-G1, LQOF-G2 and LQOF-G6), IL-12 (LQOF-G1), NO (LQOF-G1, LQOF-G2 and LQOF-G6) and ROS (LQOF-G1, LQOF-G2 and LQOF-G6), and decreased anti-inflammatory cytokines in the Th2 response, such as IL-10 (LQOF-G1 and LQOF-G2) (Figure 2). Furthermore, in the absence of infection, there were no significant changes in the levels of cytokines measured under the experimental conditions. The lack of effects on innate immunity in cells not stimulated with *Leishmania* sp. was observed in other studies and is considered a selectivity parameter in parasite control [18].

In fact, it has been proposed [21] that proteases can be directed by degrons occurring in cytokines and transcription factors of *Mus musculus*, indicating that the parasites enzymatic activity may influence Th1 and Th2 immune response profiles. Therefore, assessed guanidines could inhibit protease action and influence the macrophage immune response. Furthermore, it is plausible to propose that these compounds may influence the level of macrophage infection by inhibiting parasite arginase since arginine uptake and arginase activity are important in *Leishmania* spp. infection [22].

### 2.5. Organ Toxicity

The inotropic and chronotropic effects of compounds LQOF-G1, LQOF-G2, LQOF-G7, LQOF-G32, and LQOF-G36 were studied in isolated heart muscle preparations from guinea pigs within a concentration range of 3 to 100 µmol/L. LQOF-G1, LQOF-G7, and LQOF-G32 caused a slight decrease in heart rate up to a concentration of 100 µmol/L, while LQOF-G2 and LQOF-G36 significantly reduced heart rate activity with EC_50_ values of 69 and 38 µmol/L, respectively (Figure S14). Contractility in the papillary muscle was not affected by concentrations up to 100 µmol/L for all the compounds studied (Figure S15).

The vasodilator effect was examined in aortic and pulmonary artery rings, and the spasmolytic effect was assessed in terminal ilea. LQOF-G2 and LQOF-G36 induced a vasodilator effect in the aorta with EC_50_ values of 16 and 29 µmol/L, respectively (Figure S16), and in the pulmonary artery with EC_50_ values of 48 and 23.5 µmol/L (Figure S17). The spasmolytic effect on terminal ilea was concentration-dependent for all compounds except LQOF-G32, causing relaxation of the KCl-induced contraction force (fc).

The guanidine derivatives LQOF-G1, LQOF-G2, LQOF-G7, LQOF-G32, and LQOF-G36 demonstrated a decrease in contraction force in the terminal ileum, with LQOF-G36 presenting a spasmolytic effect (Figure S18). LQOF-G2 did not affect the heart muscle preparations, and its vasodilator activity on aortic rings was not significant. However, there was a decrease in arteria pulmonalis ring contraction force.

Guanidine derivatives are selective iNOS inhibitors, and high doses can partially inhibit the constitutive eNOS isoform [23,24,25,26]. This effect might be due to the vasodilation of the pulmonary artery. In terminal ilea, a significant spasmolytic effect was detected with an IC_50_ value of 14 µmol/L. This might be due to the antihistaminergic effects of guanidine derivatives [25].

### 2.6. Cell Toxicity

The normalized cell viability of three different cell lines treated with LQOF-G1, LQOF-G2 and LQOF-G32 (Table 2) were assessed at a concentration range of 1.56–500 µmol/L. No toxic effects on cell viability were observed, even at the highest concentration values. This demonstrated an absence of cytotoxicity for the compounds against all three cell lines. Similar results were obtained for LQOF-G6 [13]. The cell toxicity graphs can be found in Supplementary Material (Figures S19–S21).

## 3. Conclusions

The potential of guanidine compounds as a drug for *L. (V.) braziliensis* infection was explored here since these compounds can affect the parasite viability. It is important to keep in mind that further studies need to be performed since these compounds may become leishmaniasis drugs if they are potent against the parasite in the disease context without affecting the host.

Furthermore, the promising efficacy and safety profiles of these compounds suggest that they are viable candidates for further research towards developing new leishmaniasis treatments. These findings indicate that guanidine compounds are progressing along the Technology Readiness Level continuum, currently in the analytical and laboratory study phase, and moving toward the proof-of-concept stage (https://www.excellenting.com/drug_discovery_trl/, accessed on 3 July 2023). This includes conducting in vivo evaluations of their antileishmanial activity, a crucial step in their development.

## 4. Experimental Section

### 4.1. Synthesis of Guanidines

(*Z*)-*N*-benzoyl-*N*-benzyl-*N*-(4-nitrophenyl)guanidine (LQOF-G1),(*Z*)-*N*-benzoyl-*N*-benzyl-*N*-(4-bromophenyl)guanidine (LQOF-G2),(*Z*)-*N*-benzoyl-*N*-benzyl-*N*-(4-*tert*-butylphenyl)guanidine (LQOF-G6), (*Z*)-*N*-benzoyl-*N*-benzyl-N-(4-iodophenyl)guanidine (LQOF-G7), and(*Z*)-*N*-benzoyl-*N*-(2,4-di-chlorobenzyl)-*N*-(4-bromophenyl)guanidine (LQOF-G32) were synthesized as previously reported [12,13]. LQOF-G35 and LQOF-G36 are new compounds in this series. They are reported in this study and were synthesized using the same procedure [12].

### 4.2. HRESI-(+)MS Analysis

A Shimadzu GC–MS QP2010 plus system was utilized, featuring a low-polarity Rtx-5MS column (composed of 5% diphenyl and 95% dimethyl polysiloxane) with dimensions of 30 m × 2.5 mm × 0.25 μm. The system was equipped with an electron ionization source set at 70 eV and a single quadrupole. Mass spectra were acquired through direct introduction (DI) into the MS detector block. The parameters for these analyses were as follows: interface temperature: 240 °C; ionization chamber temperature: 250 °C; solvent cutting time: 0.25 min; starting time: 0.30 min; final time: 40.0 min. The DI temperature program consisted of an initial temperature of 50 °C, followed by heating at a rate of 20 °C/min until reaching 350 °C, with a waiting time of 10 min. During analysis, for LQOF-G35, its molar mass was determined to be 408.0705 g/mol, and its purity was 99.84%. For LQOF-G36, molar mass was also determined to be 408.0705 g/mol, and its purity was 99.65%.

### 4.3. NMR Measurements

The ^1^H, ^13^C, and 2D (HMBC, HSQC) NMR spectra in solution were recorded using a Bruker AVANCE-III HD-500 MHz instrument at a temperature of 253 K, unless otherwise specified. Chemical shifts (δ) were referenced to tetramethylsilane (TMS). The analyses were conducted with 15 mg of the sample dissolved in 500 µL of CDCl_3_ with a purity of 99.8 atom % D, containing 0.03% (*v*/*v*) TMS. The ^1^H NMR data are presented as follows: chemical shifts, multiplicity (s = singlet, d = doublet, dd = double doublet, t = triplet, dt = double triplet, tt = triple triplet, qua = quartet, qu = quintet, m = multiplet, br s = broad singlet), coupling constants (J) in Hertz (Hz), and peak integrals.

### 4.4. Antileishmanial Assays

#### 4.4.1. Drug Preparation

For the in vitro tests, the substances were initially dissolved in dimethyl sulfoxide (DMSO) at a concentration of 20 mg/mL. For each test, the stock solution of each substance was further diluted in the appropriate culture medium to achieve the desired concentrations.

#### 4.4.2. In Vitro Parasite Maintenance and Amastigogenesis

*Leishmania (Viannia) braziliensis* (MHOM/BR/1975/M2903) was utilized in this study. The promastigote forms of these parasites were maintained in vitro in Schneider’s insect medium pH 7.2, supplemented with 20% fetal bovine serum (FBS) and 1% antibiotic solution (100 U/mL penicillin and 100 μg/mL streptomycin) at a temperature of 26 ± 1 °C in a Biochemical Oxygen Demand (BOD) incubator (Eletrolab EL202, São Paulo, Brazil), with regular aeration and weekly subculturing [27].

In vitro amastigogenesis was performed by transformation from promastigotes at the stationary phase of growth to obtention axenic amastigotes. This process involved altering the culture conditions to Schneider’s medium with a pH of 5.5, supplementing them with 5% FBS, and incubating them at a temperature of 32 °C [17].

#### 4.4.3. Murine Macrophage Cultures

Murine macrophage cell line RAW 264.7 was used in this study. The cells were cultured in 75 cm^2^ cell culture flasks (Corning Glass Workers, New York, NY, USA) using DMEM medium with a pH of 7.2, supplemented with 10% FBS and 1% antibiotic solution (containing 100 U/mL penicillin and 100 μg/mL streptomycin). The cells were maintained in an incubator at a temperature of 37 °C with 5% CO_2_. The spikes were performed after the cells reached complete confluence, characterized by the formation of a monolayer of cells around 48 h to 72 h after packaging.

#### 4.4.4. Antileishmanial Activity on Axenic Forms of *L. (V.) braziliensis*

Assessment of antileishmanial activity against promastigote and amastigote axenic forms of parasites followed the method described by Rodrigues et al. [28] and involved the following steps: *L. (V.) braziliensis* parasites were cultured in 96-well plates at a concentration of 1 × 10^6^ parasites per well. Each well contained 100 μL of supplemented Schneider medium. Serial concentrations of the test substances, ranging from 50 to 1.56 μmol/L, were prepared. Reference drugs, meglumine antimoniate (20 to 40,000 μmol/L) and amphotericin B (0.031 to 2 μmol/L) were also prepared. These concentrations were used to assess the effects of the test substances in comparison to reference drugs. The plates were incubated in a BOD incubator at 26 °C for 72 h. After the 72 h incubation period, 10 μL of MTT (5 mg/mL) was added to each well. The plates were then incubated for an additional 4 h. At the end of the 4 h incubation, formazan crystals formed by the reduction of the MTT salt were solubilized by adding 50 μL of a 10% (*w*/*v*) sodium dodecyl sulfate (SDS) solution in distilled water. Absorbance was measured using a microplate reader at a wavelength of 540 nm (Biosystems ELx800 model, Curitiba, PR, Brazil). This measurement quantified the metabolic activity of the parasites, which is indicative of their viability and proliferation. Schneider medium containing 0.2% DMSO was used as a negative control in the tests.

#### 4.4.5. Treatment of Macrophages Infected with *L. (V.) brasiliensis*

RAW 264.7 macrophages (1 × 10^6^ cells/mL) were seeded in 24-well plates containing sterile glass slides, and approximately 500 μL of supplemented DMEM medium was added. The plates were then incubated at 37 °C with 5% CO_2_ for 4 h to facilitate cell adhesion. Following the adhesion period, promastigotes (1 × 10^7^) in the stationary growth phase were introduced into the wells for infection. The plates were then incubated for an additional 4 h to allow infection to take place. Afterward, the medium was carefully removed, and the wells were washed three times with PBS to eliminate any nonadherent cells. Fresh medium containing serial concentrations of the test substances, ranging from 0.78 to 25 μmol/L, was added to the wells. Additionally, a range of concentrations of glucantime (25 to 800 μmol/L) was included for comparison. The treatment was carried out for 72 h in an incubator at 37 °C with 5% CO_2_. At the end of the 72 h treatment period, the glass lamina was removed from each well and stained using rapid panoptic stain (Laborklin^®^, Curitiba, Paraná, Brazil). The stained samples were mounted on permanent slides using Entelan^®^ (Sigma-Aldrich, St. Louis, MO, USA), and antileishmanial activity was assessed by measuring the infection rate and counting the number of amastigotes per macrophage. Quantification was performed using light microscopy at a magnification of 1000×, and a total of 300 macrophages per lamina were evaluated. As a negative control, half DMEM containing 0.2% DMSO was used [29].

### 4.5. Immunological Assays

#### 4.5.1. Analysis of Cytokine Production

To assess the production of cytokines TNF-α and IL-12 (associated with Th1 response), as well as IL-10 and IL-6 (associated with Th2 response), in the supernatants of the macrophage infection assay, the sandwich enzyme-linked immunosorbent assay (ELISA) method was employed, following the manufacturer’s instructions (eBioscience^TM^, San Diego, CA, USA). Initially, capture antibodies specific to TNF-α, IL-6, IL-10, and IL-12 were sensitized in ELISA plates (NUNC-ImmunoTM, Sigma-Aldrich, St. Louis, MO, USA). The plates were incubated at 4 °C for 18 h. After the incubation period, all wells were washed with a solution containing 0.05% PBS and Tween 20 (Sigma-Aldrich, St. Louis, MO, USA). A blocking solution containing 10% FBS in PBS was added to the wells and incubated for 1 h. Subsequently, the wells were washed again. The supernatant obtained from the macrophage infection assay was added to the respective wells. Known concentrations of recombinant cytokines (TNF-α, IL-10, IL-6, and IL-12) were also added to the plate in parallel, covering a range from high to low concentrations. The plates were maintained at 4 °C for another 18 h. Following this incubation, additional washes were performed. The detector complex, consisting of biotinylated detection antibodies, was added to the wells. The plates were incubated for an additional hour. After another round of washing, the enzymatic complex avidin-peroxidase was added to each well. The plates were incubated for 30 min at room temperature. The reaction was initiated by adding a substrate solution containing tetramethylbenzidine and hydrogen peroxide. This reaction was allowed to proceed for 15 min. To stop the reaction, 1N sulfuric acid was added to each well. The absorbance was measured in a plate reader at a wavelength of 450 nm. The concentration of cytokines (TNF-α, IL-10, IL-6, and IL-12) in the supernatants was determined by interpolation from the standard curve [28].

#### 4.5.2. Evaluation of Nitric Oxide Synthesis Induction

To evaluate the induction of nitric oxide (NO) synthesis, the following procedure was conducted: In 96-well plates, 100 μL of the supernatants obtained from the macrophage infection assays by *L. (V.) braziliensis* were placed. An equal volume of Griess^®^ reagent (obtained from Sigma-Aldrich, St. Louis, MO, USA) was added to each well. In parallel, the same procedure was carried out using known concentrations of sodium nitrite (NaNO_2_, obtained from Vertec Química Fina, Duque de Caxias, RJ, Brazil) ranging from 125 to 2 μmol/L, which were prepared in RPMI medium. This was performedto generate a standard curve. The plates were then incubated at room temperature for 10 min. Subsequently, the absorbance of the samples was measured in a plate reader at a wavelength of 545 nm. The concentration of nitrite in the supernatants was determined by interpolating the absorbance values onto the standard curve [30].

#### 4.5.3. Determination of Reactive Oxygen Species (ROS) Production

To quantify the levels of ROS generated by *L. (V.) braziliensis*-infected RAW 264.7 macrophages, the following procedure was employed: RAW 264.7 macrophages (1 × 10^5^ cells per well) were added to a 96-well plate, with each well containing 100 μL of supplemented DMEM medium. These cells were allowed to incubate for 3 h at 37 °C with 5% CO_2_ to promote cell adhesion. Subsequently, the macrophages were infected with *L. (V.) braziliensis* at a ratio of 10:1 (promastigotes to macrophages) and incubated for 4 h. After the infection period, the cells were plated and incubated with various concentrations of test substances, ranging from 3.12 to 25 μmol/L. This incubation was carried out at 37 °C with 5% CO_2_ for duration of 72 h. Following the incubation period, 10 µL of H_2_DCFDA dye was added to each well, resulting in a final concentration of 20 µmol/L. The cells were then incubated at 37 °C in the dark for 30 min. The fluorescent intensity was measured using a fluorescence plate reader (FLx800) at an excitation wavelength of 485 nm and an emission wavelength of 528 nm. The fold change in ROS levels was calculated by comparing the results to a normal control [30].

### 4.6. Toxicity

#### 4.6.1. Experiments on Isolated Tissue Preparations

Guinea pigs of either sex weighing between 250 or 380 g were utilized in this study. The animals were housed in a controlled environment in an air-conditioned room, maintaining a temperature of 22 to 24 °C and a relative humidity of 50% to 60%. A 12 h light–dark cycle was maintained.

On the day of the experiments, the animals were euthanized via cervical dislocation. The heart, aorta, pulmonary artery, and ileum were surgically removed and immersed in Krebs–Henseleit solution, with the following composition: NaCl 144.9 mM, KCl 4.73 mM, CaCl_2_ 3.2 mM, MgSO_4_ 1.18 mM, NaHCO_3_ 24.9 mM, KH_2_PO_4_ 1.18 mM, and glucose 10 mM, with the pH maintained at 7.2–7.4. This solution was continuously aerated with a mixture of 95% O_2_ and 5% CO_2_.

Papillary muscles were isolated from the right ventricle of the heart and meticulously cleared of Purkinje fibers to prevent spontaneous activity. To ensure adequate oxygen supply, only muscles with a diameter less than 0.87 mm were used. The right atrium was also dissected for assessment of chronotropic activity. Both the aorta and pulmonary artery were cleaned and cut into 5 mm rings, while the ileum was sectioned into pieces measuring 1 to 2 cm.

One end of the dissected tissues was securely tied with silver wire for attachment to a tissue holder, while the other end was connected to a force transducer (Transbridge™, 4-Channel Transducer Amplifier, World Precision Instruments, Sarasota, FL, USA). For contractile measurements, the terminal ileum was stimulated with 60 mM KCl, and the pulmonary artery and aorta rings were exposed to a 90 mM KCl solution. Papillary muscles were electrically stimulated using an Anapulse Stimulator Model 301-T and an Isolation Unit Model 305-1 (WPI, Hamden, CT, USA) with rectangular pulses of 3 ms duration at a frequency of 1 Hz. The amplitude of the stimulation pulse was maintained at 10% above the threshold level. To optimize contractility, a constant resting tension was applied throughout the experiment: 3.9 mN for papillary muscle, 4.9 mN for terminal ileum, 10.4 mN for the right atrium, and 19.6 mN for aorta and pulmonary artery rings.

Following a 30 min control period, different concentrations of test compounds were cumulatively added to the bath solution every 45 min until a stable effect was achieved. The responses were recorded using a chart recorder (BD 112 Dual Channel, Kipp &Zonen, PO Box 507, 2624 BC, Delft, Holland) for subsequent evaluation. Stock concentrations of the test compounds were prepared using DMSO. To account for any potential DMSO effect, control experiments were conducted with the solvent alone, and the observed effects were subtracted from the responses elicited by the test compounds.

#### 4.6.2. Cell Toxicity

In this study, we utilized the RAW 264.7 macrophages line (obtained from ATCC: code TIB-71^™^),human embryonic kidney cell line HEK-293 (obtained from ATCC: code CRL-1573^™^)and two human cancer cell cultures: the colon carcinoma cell line CaCo-2 and the adenocarcinomic human alveolar basal epithelial cell A549 (also obtained from ATCC: codesHTB-37^™^ and CCL-185^™^).These cell lines were cultured in Dulbecco’s modified Eagle medium (DMEM) from Gibco by Life Technologies (LifeTech Austria, Vienna, Austria). The growth medium was supplemented with 5% fetal bovine serum (FBS) from Gibco by Life Technologies and 1% penicillin-streptomycin obtained from Sigma-Aldrich (Vienna, Austria). The cells were maintained at 37 °C with 5% CO_2_ in a humidified incubator, and regular checks for mycoplasma contamination were carried out.

For experimental purposes, the cells were seeded into 96-well plates at a density of 2 × 10^4^ cells/mL, with each well containing 100 µL of the cell suspension. The cells were allowed to adhere for 24 h. Subsequently, they were exposed to 100 µL of the test compounds, which were diluted in DMEM, with concentrations ranging from 2 to 600 µmol/L. The stock solutions of these compounds were prepared in water with 10% (*v*/*v*) dimethyl sulfoxide (DMSO) and stored at 4 °C.

To assess cell viability after 72 h of exposure to the compounds, we employed a 3-(4,5-dimethylthiazol-2-yl)-2,5-diphenyltetrazolium bromide (MTT)-based vitality assay kit (EZ4U, Biomedica, Vienna, Austria) [13]. Briefly, we added 20 µL of the EZ4U assay solution to each well and incubated the plates in the dark for 2 h. Subsequently, we measured the absorbance using a microplate reader at 450 nm, with 620 nm as a reference to reduce unspecific background values. For reliability, all experiments were conducted three times with triplicate samples.

### 4.7. Statistical Analysis

The different mean concentrations for promastigotes (IC_50_) and macrophages (CC_50_) were calculated using nonlinear regression. For comparisons between groups, ANOVA analysis of variance was performed followed by Tukey’s test in a statistical program, taking the *p*-value < 0.05 as the maximum level of statistical significance.

### 4.8. Ethics Statement

Animal experiments were carried out in accordance with the recommendations of Directive 2010/63/EU of the European Parliament and of the Council of 22 September 2010 on the protection of animals used forscientific purposes.

## Figures and Tables

**Table 1:** Antileishmanial activity, cytotoxic effects against RAW 264.7 cells, and selectivity index values calculated for guanidines and reference drugs.

Compound	Macrophages RAW 264.7	Promastigotes		Axenic Amastigotes		Intramacrophage Amastigotes	
	CC_50_ µmol/L	IC_50_ µmol/L	SI	EC_50_ µmol/L	SI	EC_50_ µmol/L	SI
LQOF-G1	147.2 ± 2.0	4.6 ± 0.5	31.9	4.3 ± 0.2	34.5	3.6 ± 0.2 ^a^	40.3
LQOF-G2	163.8 ± 2.9	5.4 ± 0.8	30.4	5.0 ± 0.8	32.4	4.2 ± 0.5 ^a^	38.9
LQOF-G6	153.6 ± 2.3	5.9 ± 0.9	26.0	4.3 ± 0.7	35.6	3.9 ± 0.1 ^a^	39.5
LQOF-G7	183.2 ± 3.1	7.2 ± 0.6	25.4	6.1	30.0	8.2 ± 0.7 ^a^	22.2
LQOF-G32	275.9 ± 2.4	17.2 ± 1.0	16.0	NT	-	NT	-
LQOF-G35	231.6 ± 3.5	25.9 ± 1.6	8.9	NT	-	NT	-
LQOF-G36	NT	>50	-	NT	-	NT	-
Meglumine At	846.4 ± 13.0	19,294 ± 195.7	0.04	2166	0.4	451.5 ± 14.3 ^a^	1.9
Amphotericin B	0.5 ± 0.1	0.3 ± 0.01	2.0	0.4	1.4	0.3 ± 0.02	1.6

Data are presented as mean ± S.E.M. of three experiments carried out in triplicate. (^a^) *p* < 0.05 vs. EC_50_ from axenic amastigotes.NT—Not tested; Meglumine At—Meglumine antimoniate; CC_50_—half-maximal cytotoxic concentration; IC_50_—half-maximal inhibitory concentration; EC_50_—half-maximal effective concentration; SI—selective index. SI = CC_50_/IC_50_ or EC_50_.

**Table 2:** Toxicity results for LQOF-G1, LQOF-G2, and LQOF-G32.Each measurement was performed in triplicate and the average value is reported.

Cell Viability (%)									
Concentrations (µmol/L)	LQOF-G1			LQOF-G2			LQOF-G32		
	CACO_2_	HEK	A549	CACO_2_	HEK	A549	CACO_2_	HEK	A549
2.34	106 ± 8	99 ± 10	114 ± 7	106 ± 3.6	104 ± 2.9	106 ± 6.3	138 ± 8.4	93 ± 5.3	109 ± 9.7
4.6875	109 ± 5	104 ± 11	97 ± 10.7	109 ± 13.6	109 ± 9.6	109 ± 5.5	123 ± 11.4	95 ± 12.7	157 ± 10.6
9.375	109 ± 7	125 ± 8.1	103 ± 8.8	109 ± 14.7	103 ± 13.5	102 ± 10.5	117 ± 7.3	89 ± 7.9	165 ± 8.1
18.75	109 ± 10	125 ± 9.4	103 ± 5.7	103 ± 5.7	108 ± 8.6	89 ± 5.7	116 ± 9.7	94 ± 8.9	121 ± 3.6
37.5	97 ± 13	100 ± 11	101 ± 8.3	97 ± 4.9	96 ± 2.9	99 ± 5.2	79 ± 6.4	91 ± 6.9	118 ± 9.9
75	99 ± 7	115 ± 7	92 ± 9.3	99 ± 6.4	99 ± 8.5	96 ± 7.7	53 ± 14.8	91 ± 13.9	115 ± 17.5
150	107 ± 3	111 ± 5	100 ± 6.5	107 ± 5.3	145 ± 3.3	109 ± 4.3	45 ± 10.4	94 ± 12.7	111 ± 15.5
300	103 ± 10	117 ± 6	110 ± 7.2	103 ± 6.6	123 ± 7.1	111 ± 9.3	45 ± 15.9	118 ± 13.9	106 ± 13.8
600	119 ± 5	115 ± 10	90 ± 8.3	119 ± 8.8	133 ± 8.4	110 ± 7.9	66 ± 12.3	185 ± 10.1	95 ± 10.4

## Data Availability

Data is contained within the article.

## Supplementary Materials

The following supporting information can be downloaded at: https://www.mdpi.com/article/10.3390/biom14010026/s1, Figure S1: ^1^H NMR spectrum of LQOF-G36 at 298K in CDCl_3_; Figure S2: ^13^C NMR spectrum of LQOF-G36 at 298K in CDCl_3_; Figure S3: EI-MS (70 eV) spectrum of compound LQOF-G36 is displayed below (*m/z* 407 M^+^ and *m/z* 105 (100%)); Figure S4: HPLC-UV analysis of LQOF-G36N; Figure S5: HRESI-(+) MS analysis of LQOF-G36; Figure S6: HRESI-(+) MS/MS analysis of LQOF-G36; Figure S7: Effects of LQOF-G1, LQOF-G2, LQOF-G6, and LQOF-G7 on the survival index of amastigotes internalized in macrophages at 72 h of exposure. RAW 264.7 macrophages were infected with promastigote forms of *L.* (*V.*) *braziliensis* and treated at different concentrations of LQOF-G1 (A), LQOF-G2 (B), LQOF-G6 (C) and LQOF-G7 (D). The results represent the means ± S.E.M. of three experiments performed in triplicate. (*) *p* < 0.05 vs. control; (**) *p* < 0.01 vs. control; (***) *p* < 0.001 vs. control. C = control; Figure S8: Effect of LQOF-G1 (▯), LQOF-G2 (●), LQOF-G7 (☐), LQOF-32 (■) and LQOF-G36 (▽) on the decrease of rate of activity of the right atrium. The decrease in percent is semilogarithmically plotted on the ordinate against the concentration of the compounds on the abscissa. Symbols represent the arithmetic means ± SEM of 4 experiments; Figure S9: Effect of LQOF-G1 (▯), LQOF-G2 (●), LQOF-G7 (☐), LQOF-32 (■) and LQOF-G36 (▽) on the decrease of contraction force of the papillary muscle. The decrease in percent is semilogarithmically plotted on the ordinate against the concentration of the compounds on the abscissa. Symbols represent the arithmetic means ± SEM of 4 experiments; Figure S10: Effect of LQOF-G1 (▯), LQOF-G2 (●), LQOF-G7 (☐), LQOF-32 (■) and LQOF-G36 (▽) on the decrease of contraction force of the aorta. The decrease in percent is semilogarithmically plotted on the ordinate against the concentration of the compounds on the abscissa. Symbols represent the arithmetic means ± SEM of 5 experiments; Figure S11: Effect of LQOF-G1 (▯), LQOF-G2 (●), LQOF-G7 (☐), LQOF-32 (■) and LQOF-G36 (▽) on the decrease of contraction force of the arteria pulmonalis. The decrease in percent is semilogarithmically plotted on the ordinate against the concentration of the compounds on the abscissa. Symbols represent the arithmetic means ± SEM of 5 experiments; Figure S12: Effect of LQOF-G1 (▯), LQOF-G2 (●), LQOF-G7 (☐), LQOF-32 (■) and LQOF-G36 (▽) on the decrease of contraction force of the terminal ileum. The decrease in percent is semilogarithmically plotted on the ordinate against the concentration of the compounds on the abscissa. Symbols represent the arithmetic means ± SEM of 5 experiments; Figure S13: Toxicity results for LQOF-G1. Bar graph showing the average percentage value of cell viability vs. concentration of LQOF-G1. Each measurement was performed in triplicate and the average value is reported; Figure S14: Toxicity results for LQOF-G2. Bar graph showing the average percentage value of cell viability vs. concentration of LQOF-G2. Each measurement was performed in triplicate and the average value is reported; Figure S15: Toxicity results for LQOF-G32. Bar graph showing the average percentage value of cell viability vs. concentration of LQOF-G32. Each measurement was performed in triplicate and the average value is reported; Figure S16: Immunomodulatory profile for LQOF-G1, LQOF-G2, LQOF-G6 in macrophages RAW 264.7 without infection. (A) TNF-α; (B) IL-12; (C) IL-10; (D) IL-6; (E) Nitrite and (F) reactive oxygen species (ROS) were evaluated using the supernatant of cultured RAW 267.4 macrophages. Data represent the mean ± standard error of the mean of at least. Comparison between groups was performed by *One-way* ANOVA followed by Tukey’s post-test, where ** *p <* 0.01 and *** *p <* 0.001 compared to the control. LPS—lipopolysaccharide; Table 1: Representative ^1^H and ^13^C chemical shifts (ppm) at 298 K for LQOF-G36.
