# Supplementary material for: Evidence of Guanidines Potential against Leishmania (Viannia) braziliensis: Exploring In Vitro Effectiveness, Toxicities and of Innate Immunity Response Effects
Source: Biomolecules. 2023 Dec 24;14(1):26. doi: 10.3390/biom14010026 (PMC10813298; doi:10.3390/biom14010026)
Supplement: Supplementary file 1 [file biomolecules-14-00026-s001.zip › biomolecules-2788159-supplementary.pdf]

Evidence of Guanidines Potential against Leishmania (Viannia) braziliensis: Exploring In Vitro Effectiveness, Toxicities and of Innate Immunity Response Effects

Luana Ribeiro dos Anjos 1 , Vanessa Maria Rodrigues de Souza 2, Yasmim Alves Aires Machado 2, Vitor Moreira Partite 1, Mohammed Aufy 3 , Geovane Dias Lopes 4 , Christian Studenik 3 , Carlos Roberto Alves 4, Gert Lubec 5 , Eduardo Rene Perez Gonzalez 1,* and Klinger Antonio da Franca Rodrigues 2,*

1 Fine Organic Chemistry Lab, School of Sciences and Technology, São Paulo State University (UNESP),
Presidente Prudente 19060-080, Brazil; luana.anjos@unesp.br (L.R.d.A.); vitor.partite@unesp.br (V.M.P.)
2 Infectious Disease Laboratory—LADIC, Federal University of Parnaíba Delta—UFDPar, Campus Ministro
Reis Velloso, São Benedito, Parnaíba 64202-020, Brazil; rodriguesvanessa745@ufpi.edu.br (V.M.R.d.S.);
machado03@ufpi.edu.br (Y.A.A.M.)
3 Department of Pharmaceutical Sciences, Division of Pharmacology and Toxicology, University of Vienna,
Josef Holaubek Platz 2, UZAII (2D 259), 1090 Vienna, Austria; mohammed.aufy@univie.ac.at (M.A.);
christian.studenik@univie.ac.at (C.S.)
4 Laboratório de Biologia Molecular e Doenças Endêmicas, Fundação Oswaldo Cruz, Instituto Oswaldo Cruz,
4365, Manguinhos, Rio de Janeiro 21040-900, Brazil; geovane.dl@gmail.com (G.D.L.); klvess@gmail.com (C.R.A.)
5 Department of Neuroproteomics, Paracelsus Medical University, 5020 Salzburg, Austria; gert.lubec@lubeclab.com
* Correspondence: eduardo.gonzalez@unesp.br (E.R.P.G.); klinger@ufpi.edu.br (K.A.d.F.R.)

Table of contents

1. Structural characterization for LQOF-G35 and LQOF-G36 4

2. NMR analysis for LQOF-35 and LQOF-G36 5

2.1 NMR measurements 5

Table S1. Representative ^{1}H and ^{13}C chemical shifts (ppm) at 298K for **LQOF-G35**. 5

Table S2. Representative ^{1}H and ^{13}C chemical shifts (ppm) at 298K for **LQOF-G36**. 6

2.2. NMR Spectra 7

Fig. S1. ^{1}H NMR spectrum of **LQOF-G35** at 298K in $CDCl_3$. 7

Fig. S2. ^{13}C NMR spectrum of **LQOF-G35** at 298K in $CDCl_3$. 7

Fig. S3. ^{1}H NMR spectrum of **LQOF-G36** at 298K in $CDCl_3$. 8

Fig. S4. ^{13}C NMR spectrum of **LQOF-G36** at 298K in $CDCl_3$. 8

3. EI-MS analysis 9

Fig. S5. EI-MS (70 eV) spectrum of compound **LQOF-G35** is displayed below (*m/z* 409 M^+ and *m/z* 105 (100 %)). 9

Fig. S6. EI-MS (70 eV) spectrum of compound **LQOF-G36** is displayed below (*m/z* 407 M^+ and *m/z* 105 (100 %)). 9

4. LC/MS with UV/Vis detection analysis for LQOF-G35 and LQOF-G36 10

4.1. LC/MS with UV/Vis and MS/MS results 10

Fig. S7. HPLC-UV analysis of **LQOF-G35**. 10

Fig. S8. HPLC-UV analysis of **LQOF-G36**. 11

Fig. S9. HRESI-(+) MS analysis of **LQOF-G35**. 12

Fig. S10. HRESI-(+) MS/MS analysis of **LQOF-G35**. 13

Fig. S11. HRESI-(+) MS analysis of **LQOF-G36**. 14

Fig. S12. HRESI-(+) MS/MS analysis of **LQOF-G36**. 15

5. In vitro efficacy of guanidines LQOF-G1, LQOF-G2, LQOF-G6 and LQOF-G7 against intramacrophage amastigotes 16

Fig. S13. Effects of LQOF-G1, LQOF-G2, LQOF-G6, and LQOF-G7 on the survival index of amastigotes internalized in macrophages at 72 h of exposure. RAW 264.7 macrophages were infected with promastigote forms of *L.* (*V.*) *braziliensis* and treated at different concentrations of LQOF-G1 (A), LQOF-G2 (B), LQOF-G6 (C) and LQOF-G7 (D). The results represent the means ± S.E.M. of three experiments performed in triplicate. (*) $p < 0.05$ vs. control; (**) $p < 0.01$ vs. control; (***) $p < 0.001$ vs. control. C = control. 16

7. Organ Toxicity 17

Fig. S14. Effect of LQOF-G1 (□), LQOF-G2 (●), LQOF-G7 (□), LQOF-32 (■) and LQOF-G36 (▽) on the decrease of rate of activity of the right atrium. The decrease in percent is

semilogarithmically plotted on the ordinate against the concentration of the compounds on the abscissa. Symbols represent the arithmetic means ± SEM of 4 experiments. 17

Fig. S15. Effect of LQOF-G1 (□), LQOF-G2 (●), LQOF-G7 (◻), LQOF-32 (■) and LQOF-G36 (▽) on the decrease of contraction force of the papillary muscle. The decrease in percent is semilogarithmically plotted on the ordinate against the concentration of the compounds on the abscissa. Symbols represent the arithmetic means ± SEM of 4 experiments. 17

Fig. S16. Effect of LQOF-G1 (□), LQOF-G2 (●), LQOF-G7 (◻), LQOF-32 (■) and LQOF-G36 (▽) on the decrease of contraction force of the aorta. The decrease in percent is semilogarithmically plotted on the ordinate against the concentration of the compounds on the abscissa. Symbols represent the arithmetic means ± SEM of 5 experiments. 18

Fig. S17. Effect of LQOF-G1 (□), LQOF-G2 (●), LQOF-G7 (◻), LQOF-32 (■) and LQOF-G36 (▽) on the decrease of contraction force of the arteria pulmonalis. The decrease in percent is semilogarithmically plotted on the ordinate against the concentration of the compounds on the abscissa. Symbols represent the arithmetic means ± SEM of 5 experiments. 18

Fig. S18. Effect of LQOF-G1 (□), LQOF-G2 (●), LQOF-G7 (◻), LQOF-32 (■) and LQOF-G36 (▽) on the decrease of contraction force of the terminal ileum. The decrease in percent is semilogarithmically plotted on the ordinate against the concentration of the compounds on the abscissa. Symbols represent the arithmetic means ± SEM of 5 experiments. 19

8. Cell Toxicity Graphics 20

Fig. S19. Toxicity results for LQOF-G1. Bar graph showing the average percentage value of cell viability vs. concentration of LQOF-G1. Each measurement was performed in triplicate and the average value is reported. 20

Fig. S20. Toxicity results for LQOF-G2. Bar graph showing the average percentage value of cell viability vs. concentration of LQOF-G2. Each measurement was performed in triplicate and the average value is reported. 20

Fig. S21. Toxicity results for LQOF-G32. Bar graph showing the average percentage value of cell viability vs. concentration of LQOF-G32. Each measurement was performed in triplicate and the average value is reported. 21

1. Structural characterization for LQOF-G35 and LQOF-G36

LQOF-G35 and **LQOF-G36** has been synthesized by previously reported method (Santo et al., 2019)[1]. The **LQOF-G35** and **LQOF-G36** was characterized as follows:

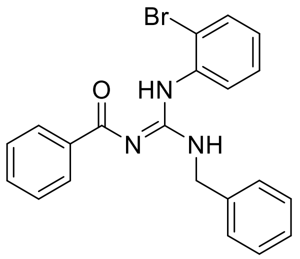

(*Z*)-*N*-benzoyl-*N*-benzyl-*N*-(2-bromophenyl)guanidine – (LQOF-G35); m.p. = 103,7 – 104,2°C; recrystallized from di-ethylether/petroleum ether (1:4); **LC-UV/MS**: 99.84%; **ESI(+)-MS** *m/z* found 408.0705, *m/z* calculated for $[C_{21}H_{18}BrN_3O + H]^+$: 408.0706; **ESI(+)-MS/MS**: $M + H – C_6H_5CONH_2]^+$ *m/z* 287.0175, $[M + H – C_{15}H_{12}N_2O]^+$ *m/z* 171.9757, $[M + H – C_{13}H_{12}BrN_2]^+$ *m/z* 122.0603; **^{1}H NMR** (500.16 MHz, $CDCl_3$) δ ppm = 12.31 (s, 1H), 8.27 (s, 2H), 7.64 (m, 1H), 7.49 (t, 1H), 7.40 (m, 4H), 7.35 (m, 3H), 7.31 (m, 1H), 7.29 (t, 1H), 7.12 (s, 1H), 4.77 (s, 2H); **^{13}C NMR** (125.765 MHz, $CDCl_3$) δ ppm = 178.02 (C=O), 158.38 (N=C), 138.29 (C), 135.26 (C), 131.38 (CH), 129.28 (2CH), 128.87 (3CH), 128.61 (2CH), 127.92 (1CH), 127.59 (3CH), 45.39 (CH_2).

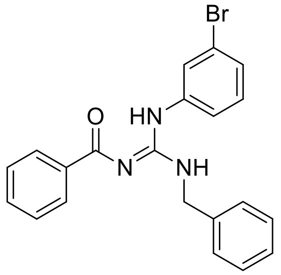

(*Z*)-*N*-benzoyl-*N*-benzyl-*N*-(3-bromophenyl)guanidine – (LQOF-G36); m.p. = 86.2 – 86.6°C; recrystallized from di-ethylether/petroleum ether (1:4); **LC-UV/MS**: 99.65%; **ESI(+)-MS** *m/z* found 408.0705, *m/z* calculated for $[C_{21}H_{18}BrN_3O + H]^+$: 408.0706; **ESI(+)-MS/MS**: $M + H – C_6H_5CONH_2]^+$ *m/z* 287.0169, $[M + H – C_{15}H_{12}N_2O]^+$ *m/z* 171.9755, $[M + H – C_{13}H_{12}BrN_2]^+$ *m/z* 122.0602; **^{1}H NMR** (500.16 MHz, $CDCl_3$) δ ppm = 12.30 (s, 1H), 8.25 (s, 2H), 7.48 (m, 3H), 7.42 (m, 1H), 7.41 (m,1H), 7.39 (m, 1H), 7.37 (m, 4H), 7.31 (m, 1H), 7.19 (s, 1H), 4.78 (s, 2H); **^{13}C NMR** (125.765 MHz, $CDCl_3$) δ ppm = 178.02 (C=O),

158.34 (N=C), 138.22 (C), 131.42 (C), 131.19 (C), 129.21 (2CH), 128.91 (3CH), 127.94 (2CH), 127.78 (1CH), 127.58 (3CH), 45.34 (CH_2).

2. NMR analysis for LQOF-35 and LQOF-G36

2.1 NMR measurements

All chemical shifts are presented below.

Table S1. Representative ^1H and ^{13}C chemical shifts (ppm) at 298K for **LQOF-G35**.

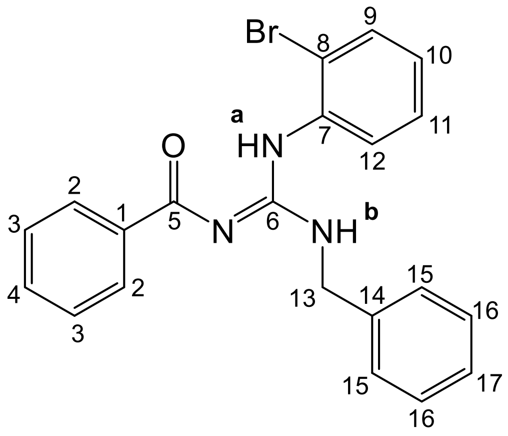

^1H chemical shifts							
**H2**	**H3**	**H4**	**H9**	**H10**	**H11**	**H12**	**H13**
8.27	7.35	7.49	7.64	7.35	7.12	7.31	4.77
**H15**	**H16**	**H17**	**Ha**				
7.40	7.40	7.29	12.31				
**^{13}C chemical shifts**							
**C1**	**C2**	**C3**	**C4**	**C5**	**C6**	**C7**	**C8**
-	129.28	128.61	131.38	178.02	158.38	138.29	-
**C9**	**C10**	**C11**	**C12**	**C13**	**C14**	**C15**	**C16**
-	127.59	128.87	-	45.39	135.26	127.59	128.87
**C17**							
127.92							

Table S2. Representative ^{1}H and ^{13}C chemical shifts (ppm) at 298K for **LQOF-G36**.

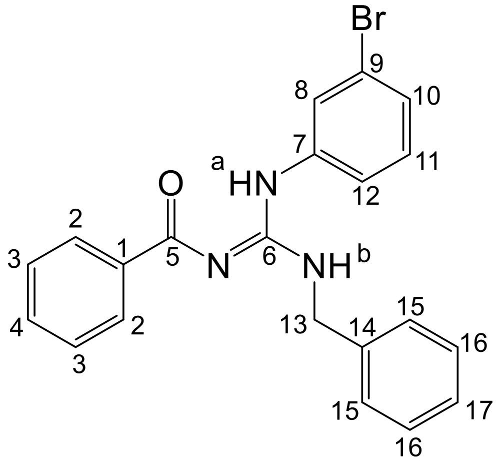

**^{1}H chemical shifts**							
**H2**	**H3**	**H4**	**H8**	**H10**	**H11**	**H12**	**H13**
8.25	7.48	7.48	7.42	7.39	7.19	7.31	4.78
**H15**	**H16**	**H17**	**Ha**				
7.37	7.37	7.41	12.30				
**^{13}C chemical shifts**							
**C1**	**C2**	**C3**	**C4**	**C5**	**C6**	**C7**	**C8**
131.42	129.21	127.94	-	178.02	158.34	131.19	-
**C9**	**C10**	**C11**	**C12**	**C13**	**C14**	**C15**	**C16**
138.22	-	128.91	127.58	45.34	-	127.58	128.91
**C17**							
127.78							

2.2. NMR Spectra

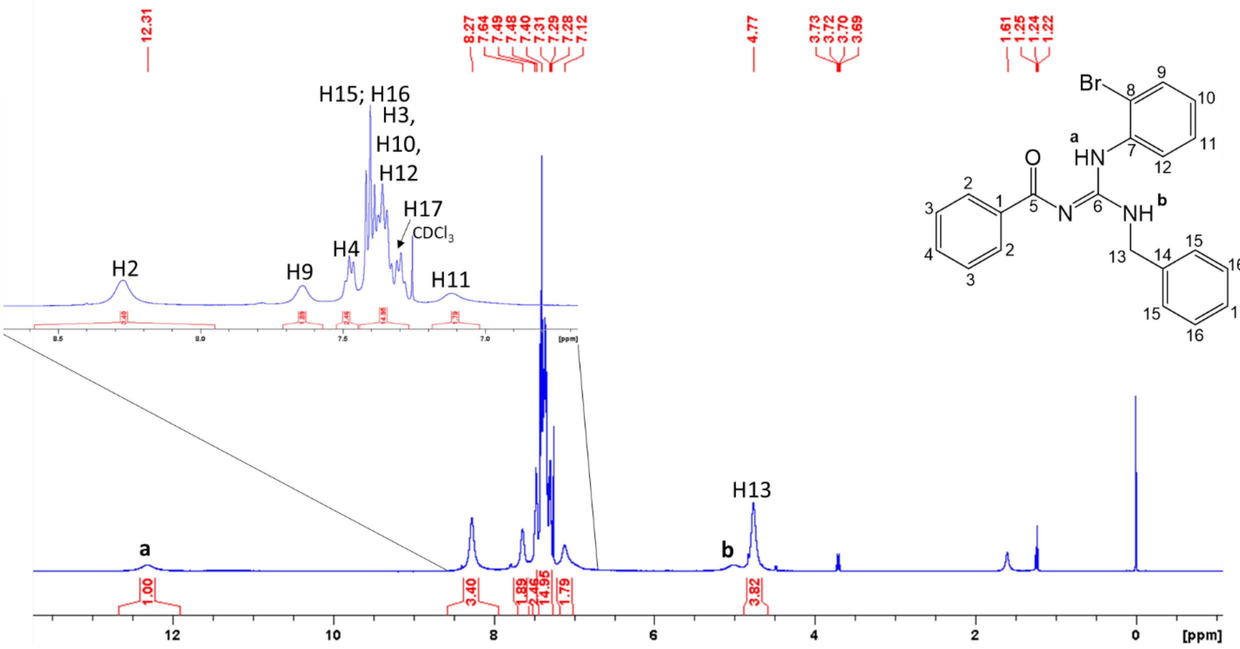

Fig. S1. ^{1}H NMR spectrum of **LQOF-G35** at 298K in $CDCl_3$.

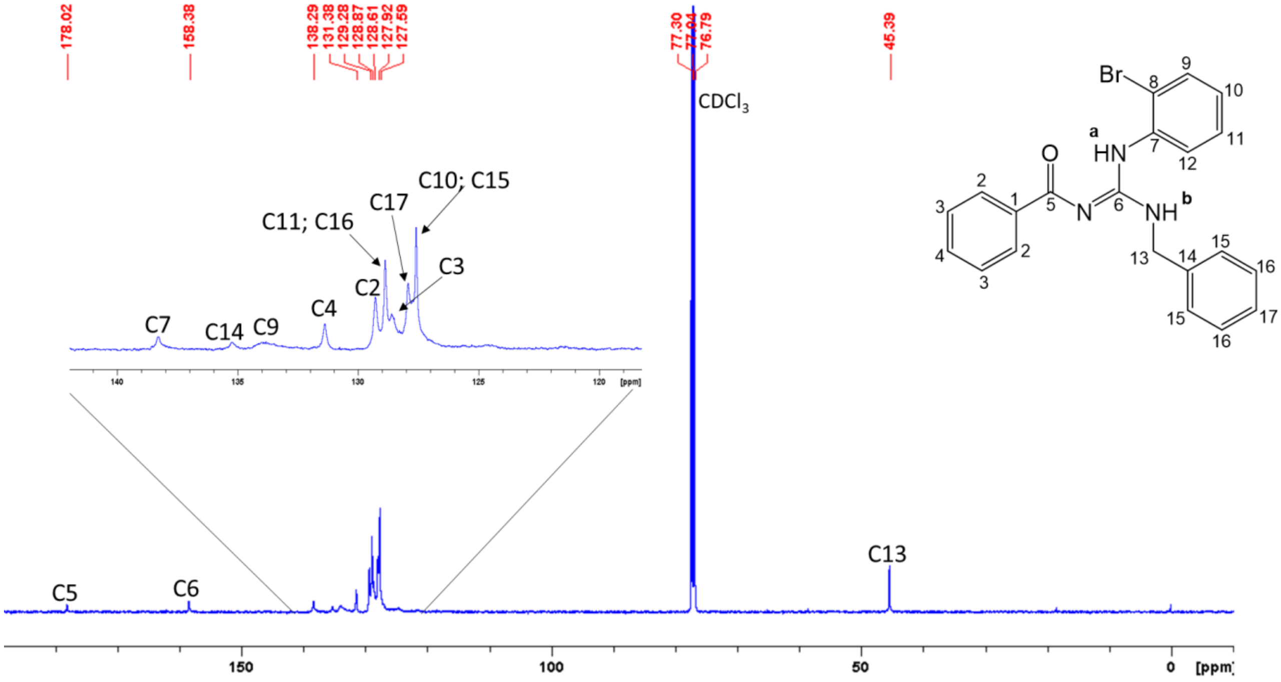

Fig. S2. ^{13}C NMR spectrum of **LQOF-G35** at 298K in $CDCl_3$.

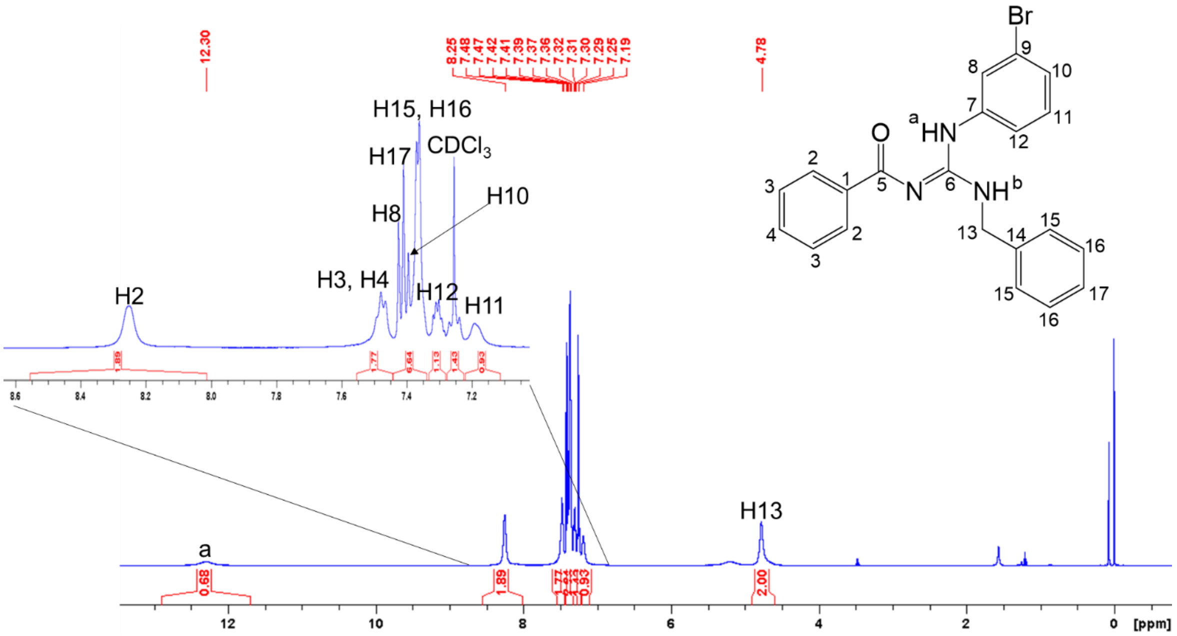

Fig. S3. ^{1}H NMR spectrum of **LQOF-G36** at 298K in $CDCl_3$.

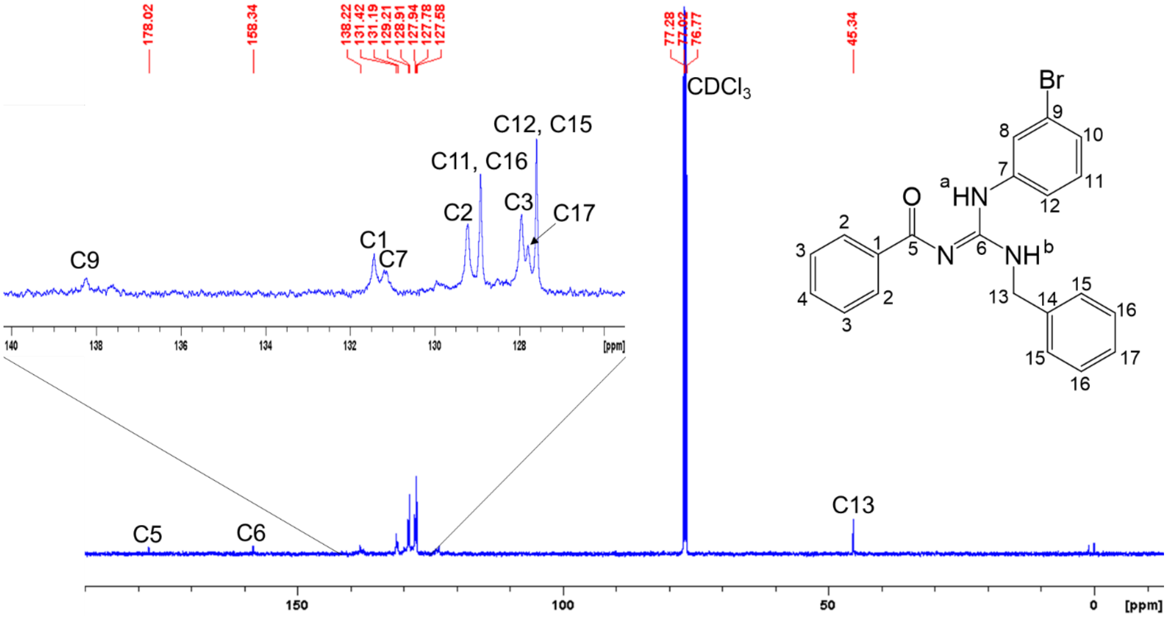

Fig. S4. ^{13}C NMR spectrum of **LQOF-G36** at 298K in $CDCl_3$.

3. EI-MS analysis

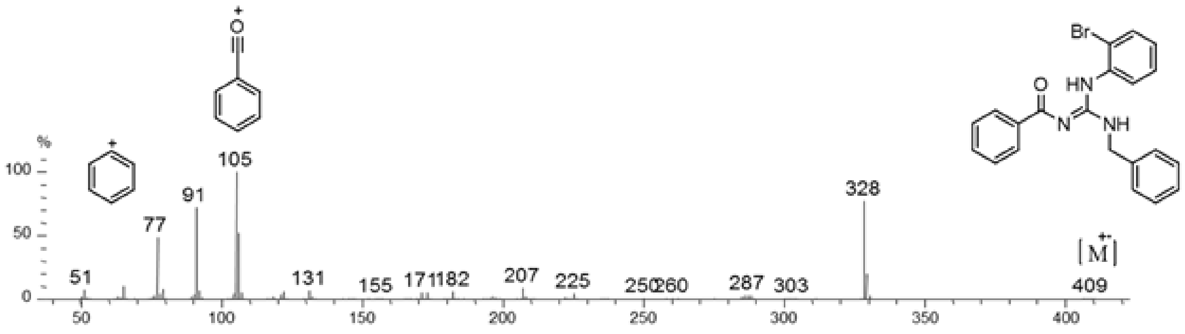

Fig. S5. EI-MS (70 eV) spectrum of compound **LQOF-G35** is displayed below (*m/z* 409 M^+ and *m/z* 105 (100 %)).

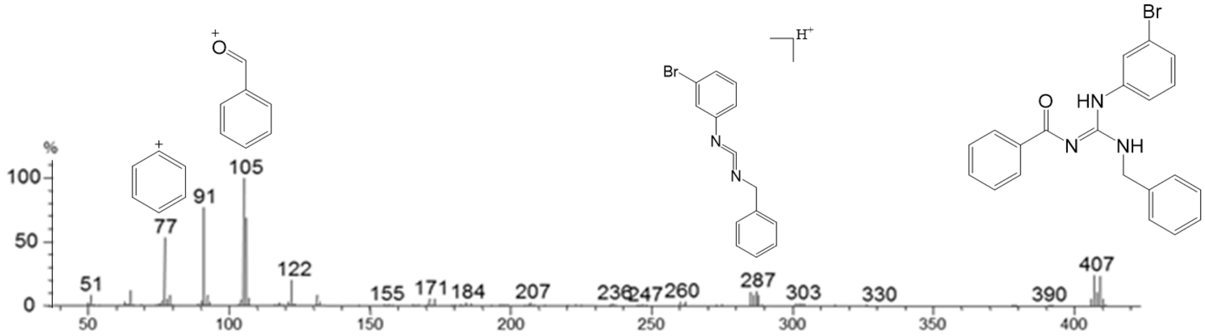

Fig. S6. EI-MS (70 eV) spectrum of compound **LQOF-G36** is displayed below (*m/z* 407 M^+ and *m/z* 105 (100 %)).

4. LC/MS with UV/Vis detection analysis for LQOF-G35 and LQOF-G36

4.1. LC/MS with UV/Vis and MS/MS results

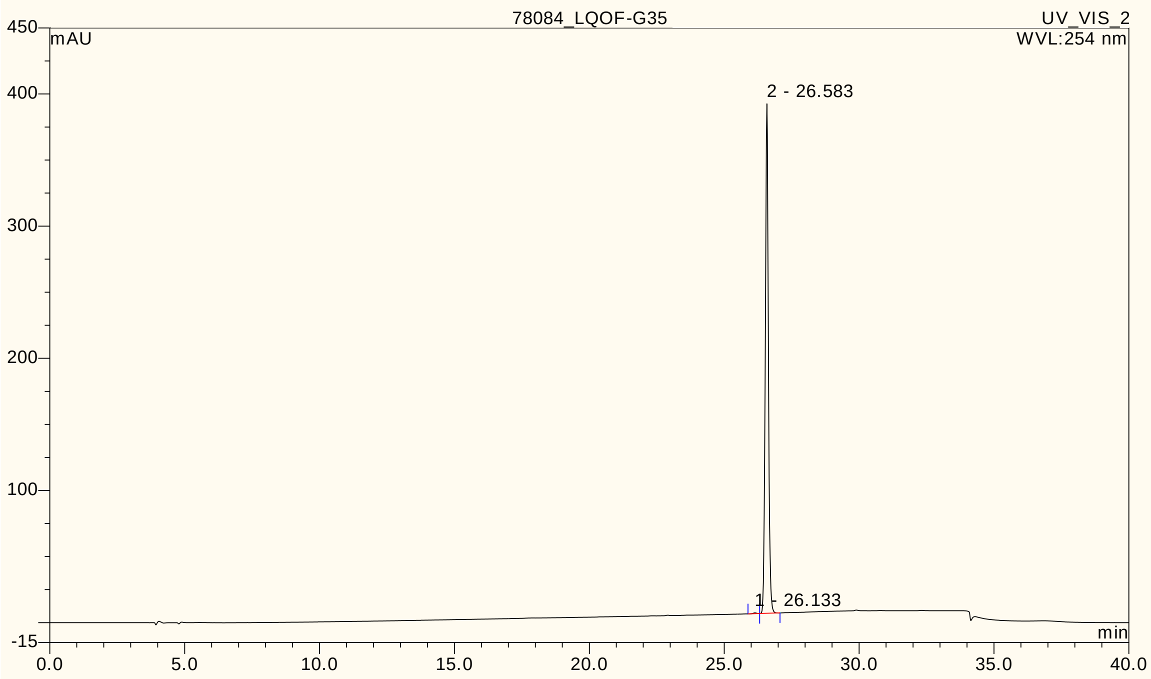

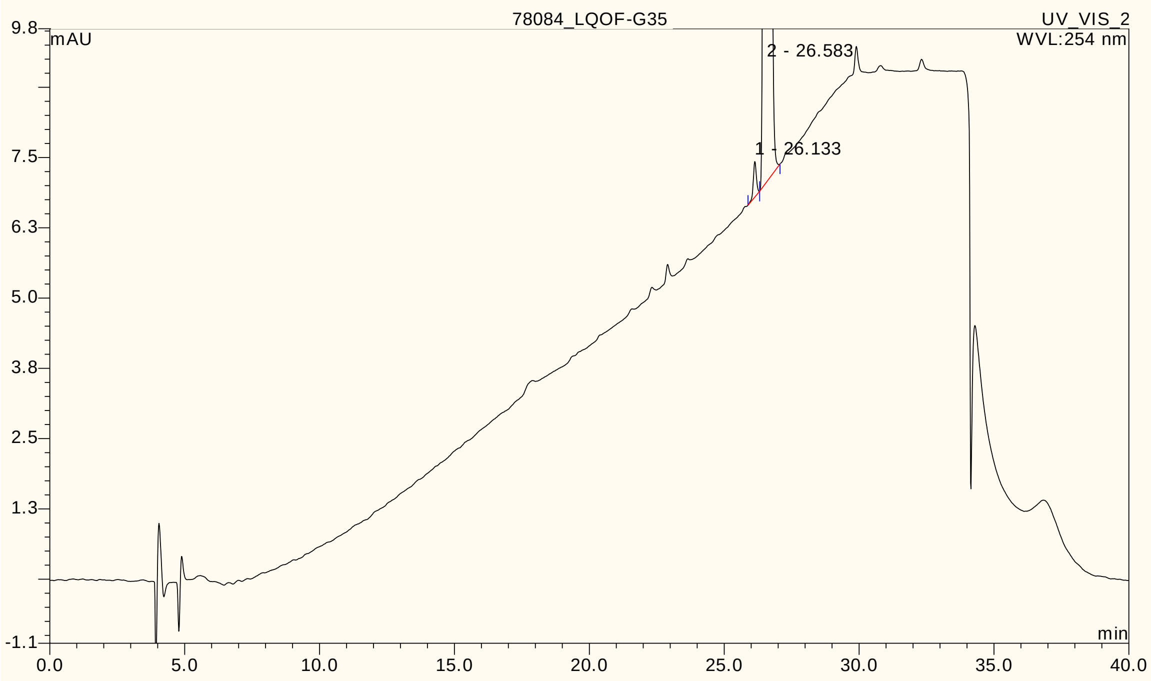

Retention Time: **26.58 min**

Relative Peak Area: **99.84 %**

Fig. S7. HPLC-UV analysis of **LQOF-G35**.

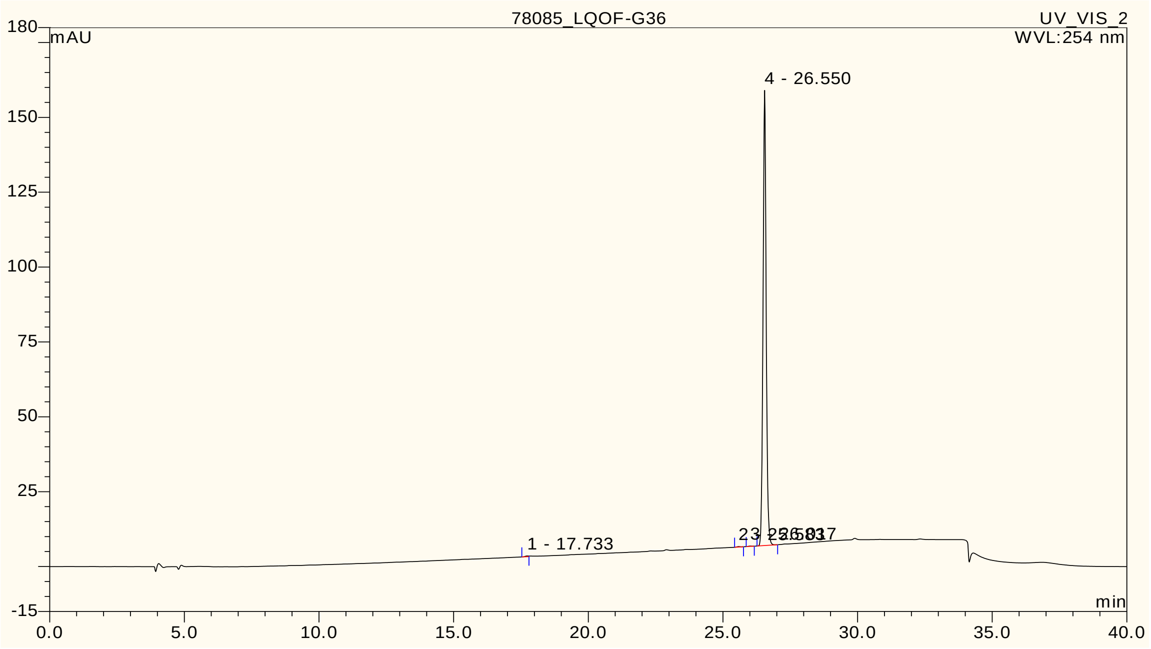

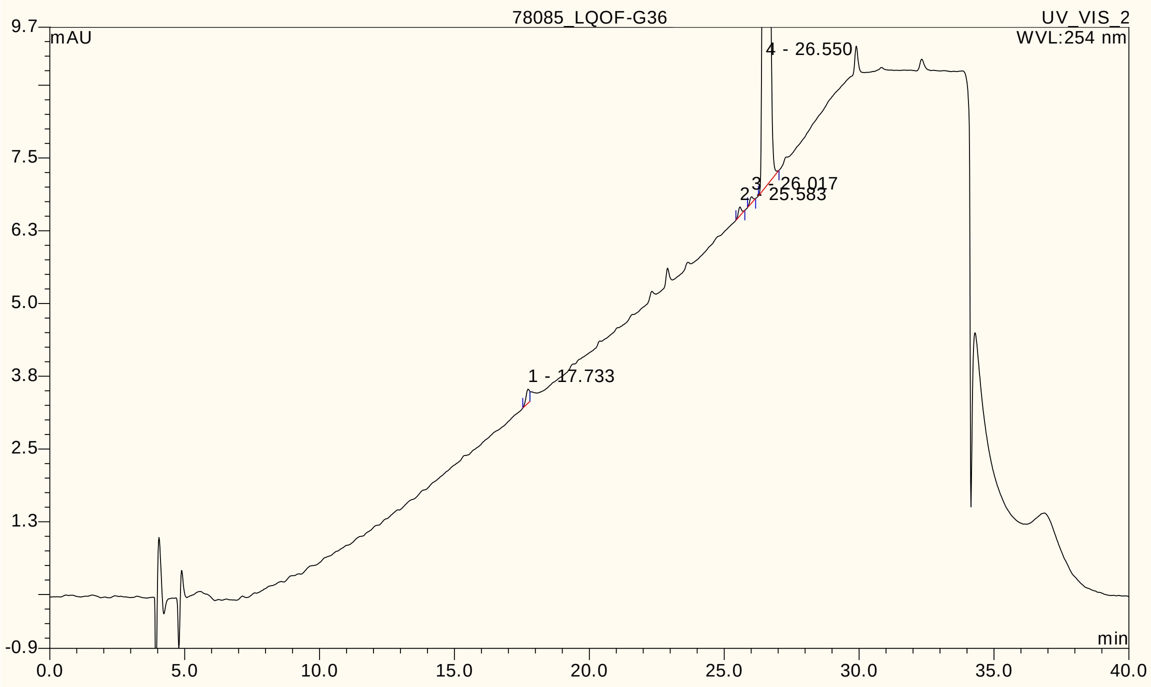

Retention Time: **26.55 min**

Relative Peak Area: **99.65 %**

Fig. S8. HPLC-UV analysis of **LQOF-G36**.

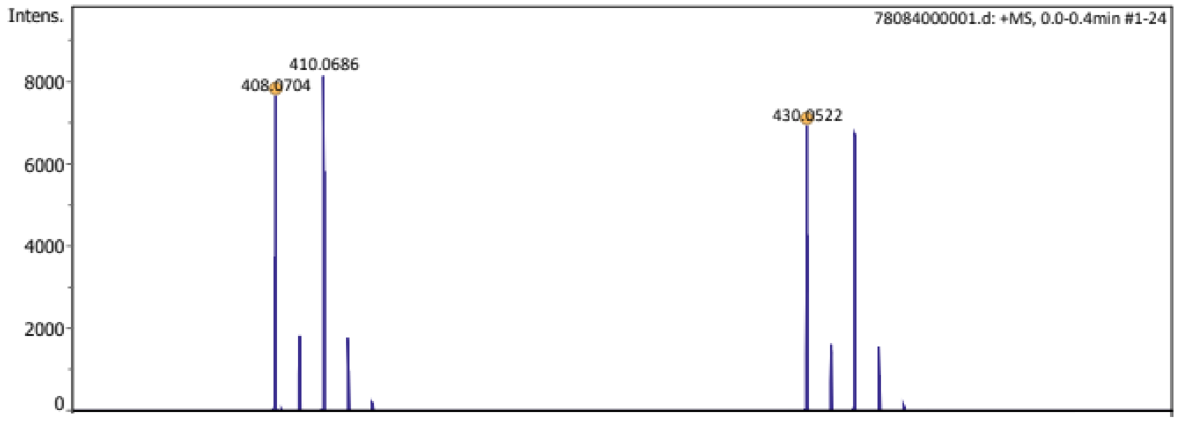

m/z 408,0704: $C_{21}H_{19}BrN_3O$

m/z 430,0522: $C_{21}H_{18}BrN_3NaO$

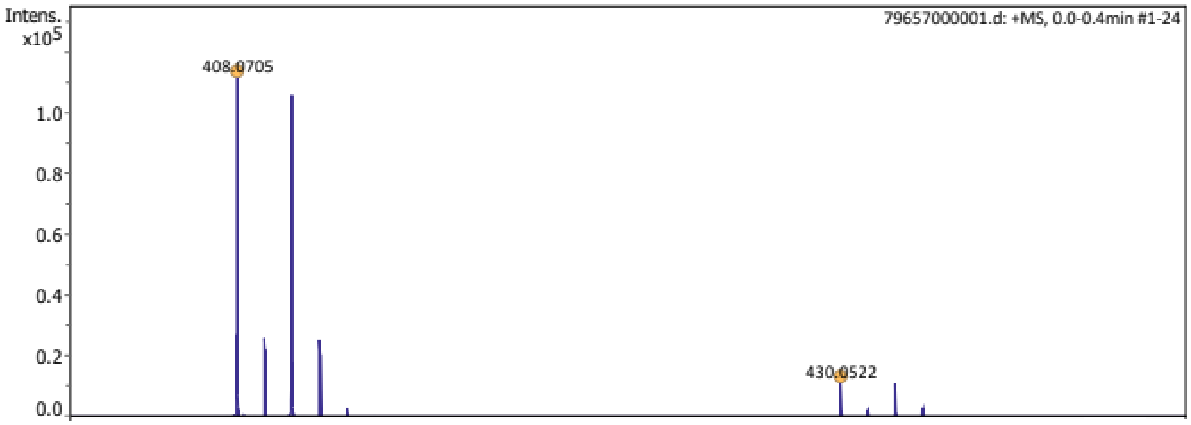

m/z 408,0705: $C_{21}H_{19}BrN_3O$

m/z 430,0522: $C_{21}H_{18}BrN_3NaO$

Fig. S9. HRESI-(+) MS analysis of **LQOF-G35**.

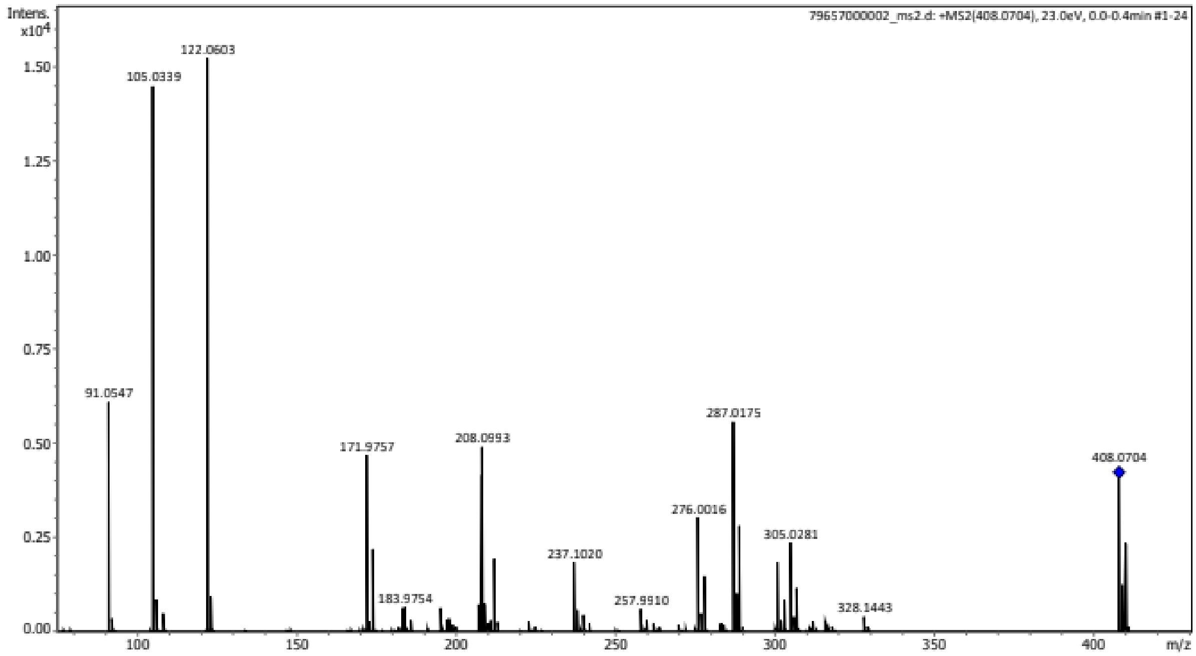

Fig. S10. HRESI-(+) MS/MS analysis of **LQOF-G35**.

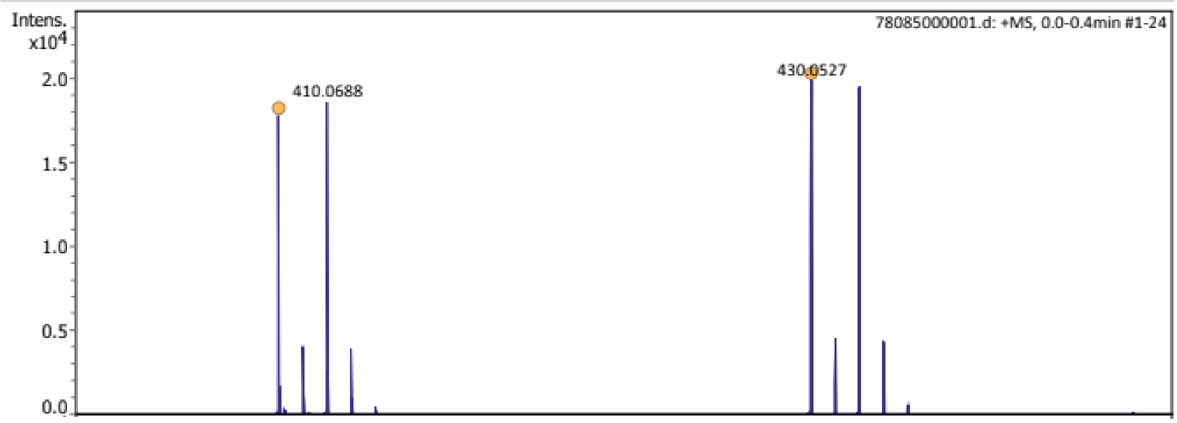

m/z 408.0707: $C_{21}H_{19}BrN_3O$

m/z 430.0527: $C_{21}H_{18}BrN_3NaO$

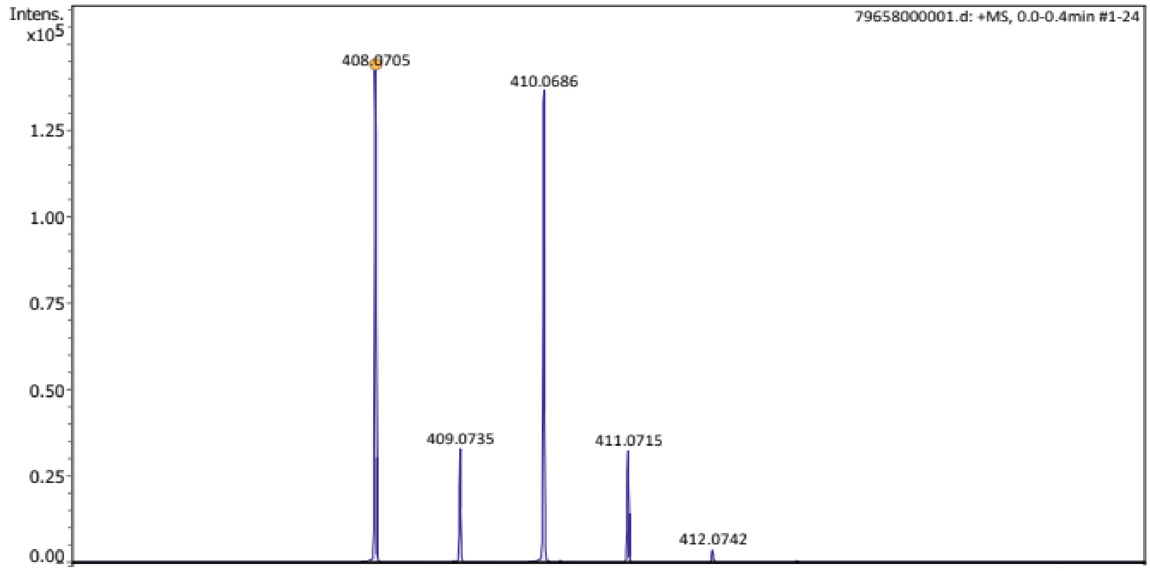

m/z 408.0705: $C_{21}H_{19}BrN_3O$

Fig. S11. HRESI-(+) MS analysis of **LQOF-G36**.

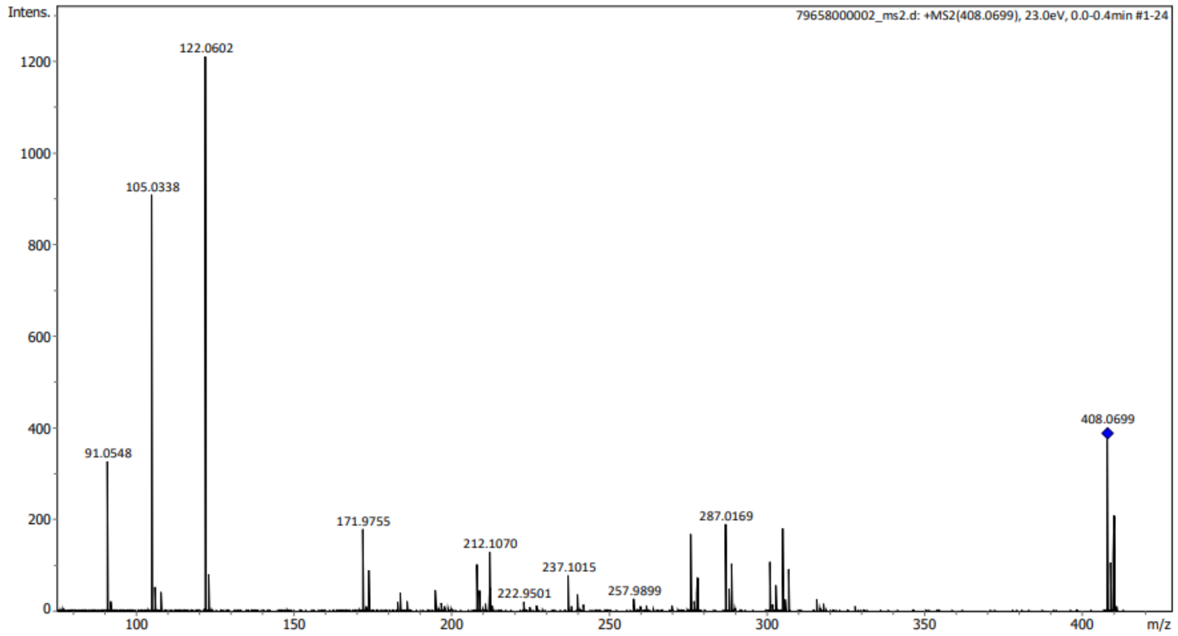

Fig. S12. HRESI-(+) MS/MS analysis of **LQOF-G36**.

5. In vitro efficacy of guanidines LQOF-G1, LQOF-G2, LQOF-G6 and LQOF-G7 against intramacrophage amastigotes

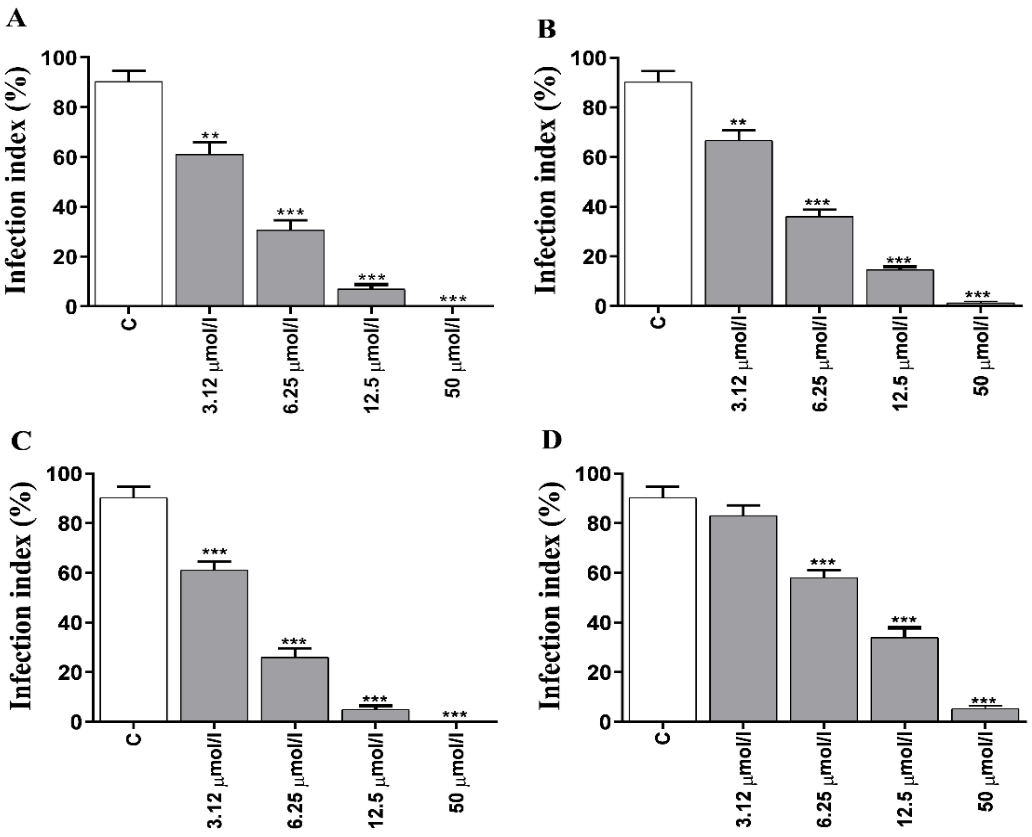

Fig. S13. Effects of LQOF-G1, LQOF-G2, LQOF-G6, and LQOF-G7 on the survival index of amastigotes internalized in macrophages at 72 h of exposure. RAW 264.7 macrophages were infected with promastigote forms of *L.* (*V.*) *braziliensis* and treated at different concentrations of LQOF-G1 (A), LQOF-G2 (B), LQOF-G6 (C) and LQOF-G7 (D). The results represent the means ± S.E.M. of three experiments performed in triplicate. (*) $p < 0.05$ vs. control; (**) $p < 0.01$ vs. control; (***) $p < 0.001$ vs. control. C = control.

7. Organ Toxicity

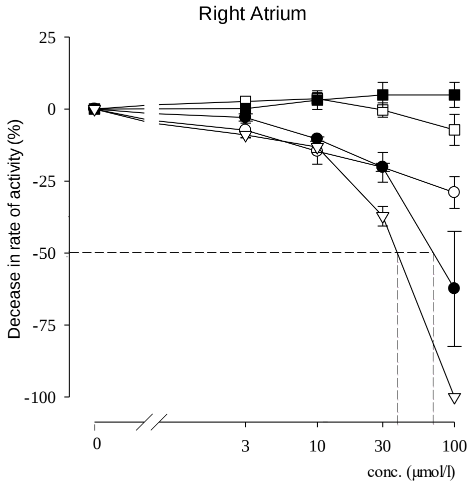

Fig. S14. Effect of LQOF-G1 (□), LQOF-G2 (●), LQOF-G7 (□), LQOF-32 (■) and LQOF-G36 (▽) on the decrease of rate of activity of the right atrium. The decrease in percent is semilogarithmically plotted on the ordinate against the concentration of the compounds on the abscissa. Symbols represent the arithmetic means ± SEM of 4 experiments.

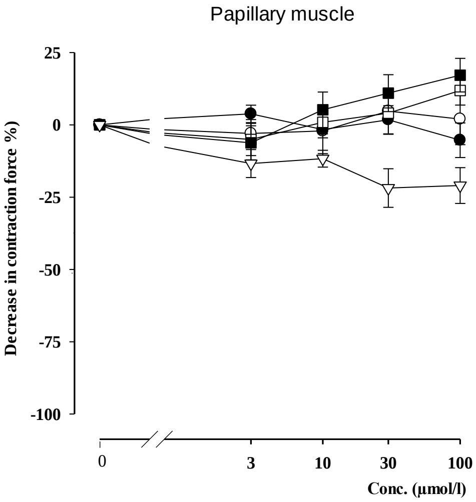

Fig. S15. Effect of LQOF-G1 (□), LQOF-G2 (●), LQOF-G7 (□), LQOF-32 (■) and LQOF-G36 (▽) on the decrease of contraction force of the papillary muscle. The decrease in percent is semilogarithmically plotted on the ordinate against the concentration of the compounds on the abscissa. Symbols represent the arithmetic means ± SEM of 4 experiments.

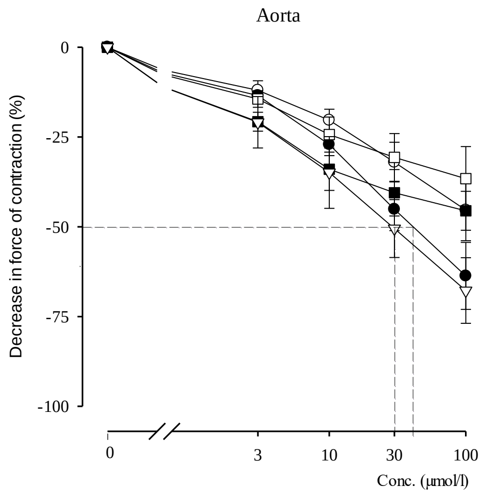

Fig. S16. Effect of LQOF-G1 (□), LQOF-G2 (●), LQOF-G7 (□), LQOF-32 (■) and LQOF-G36 (▽) on the decrease of contraction force of the aorta. The decrease in percent is semilogarithmically plotted on the ordinate against the concentration of the compounds on the abscissa. Symbols represent the arithmetic means ± SEM of 5 experiments.

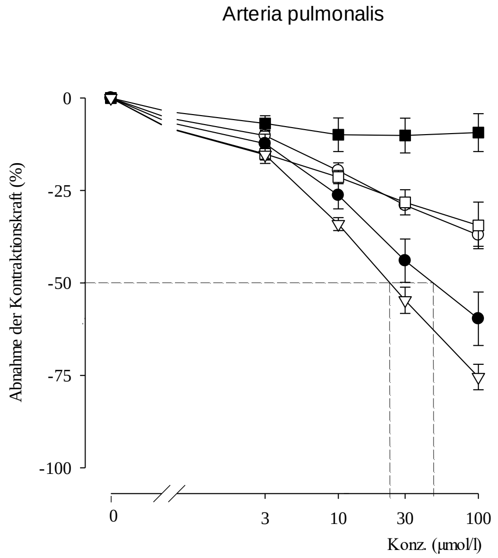

Fig. S17. Effect of LQOF-G1 (□), LQOF-G2 (●), LQOF-G7 (□), LQOF-32 (■) and LQOF-G36 (▽) on the decrease of contraction force of the arteria pulmonalis. The decrease in percent is semilogarithmically plotted on the ordinate against the concentration of the compounds on the abscissa. Symbols represent the arithmetic means ± SEM of 5 experiments.

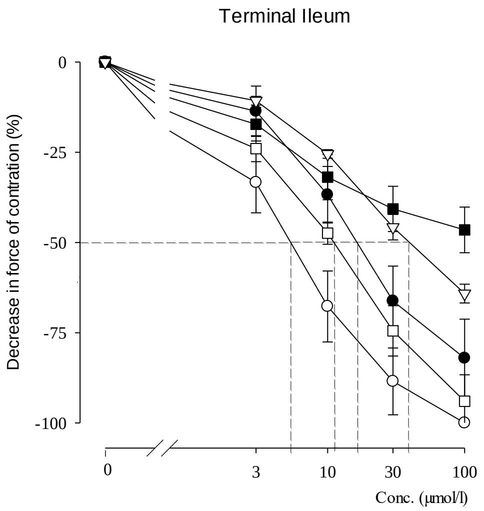

Fig. S18. Effect of LQOF-G1 (□), LQOF-G2 (●), LQOF-G7 (□), LQOF-32 (■) and LQOF-G36 (▽) on the decrease of contraction force of the terminal ileum. The decrease in percent is semilogarithmically plotted on the ordinate against the concentration of the compounds on the abscissa. Symbols represent the arithmetic means ± SEM of 5 experiments.

8. Cell Toxicity Graphics

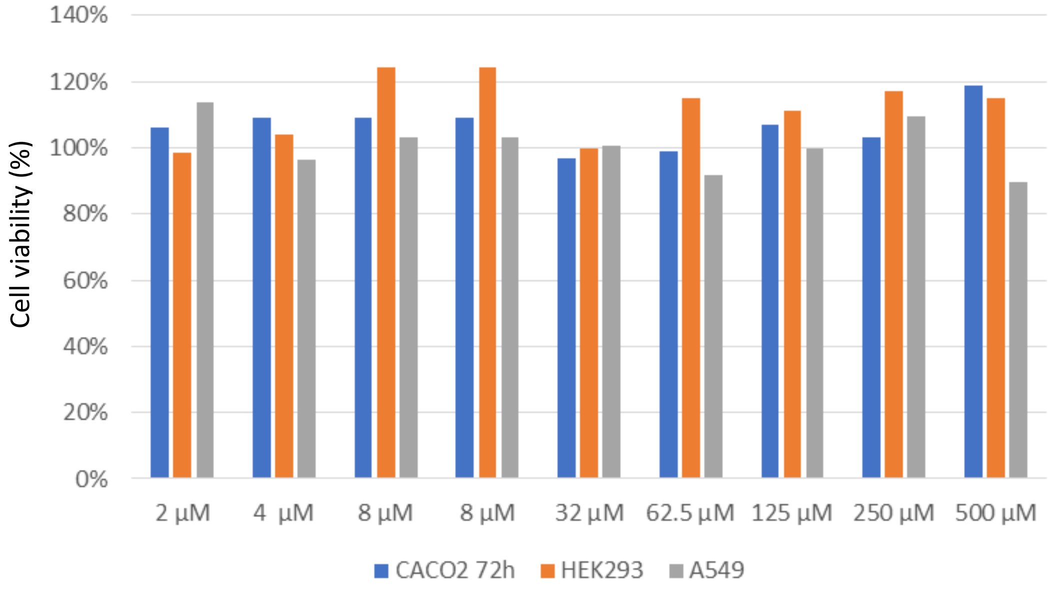

Fig. S19. Toxicity results for LQOF-G1. Bar graph showing the average percentage value of cell viability vs. concentration of LQOF-G1. Each measurement was performed in triplicate and the average value is reported.

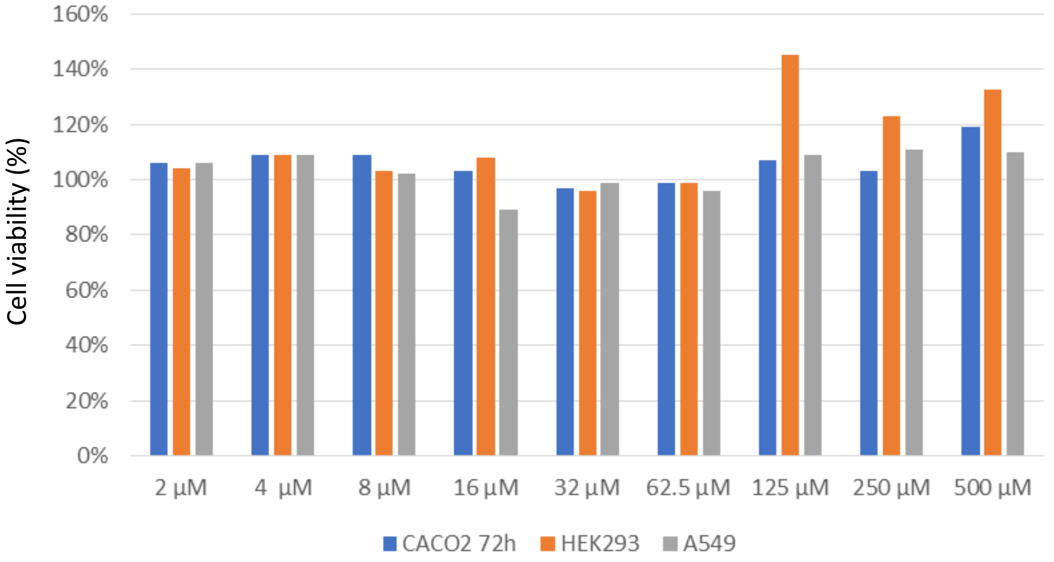

Fig. S20. Toxicity results for LQOF-G2. Bar graph showing the average percentage value of cell viability vs. concentration of LQOF-G2. Each measurement was performed in triplicate and the average value is reported.

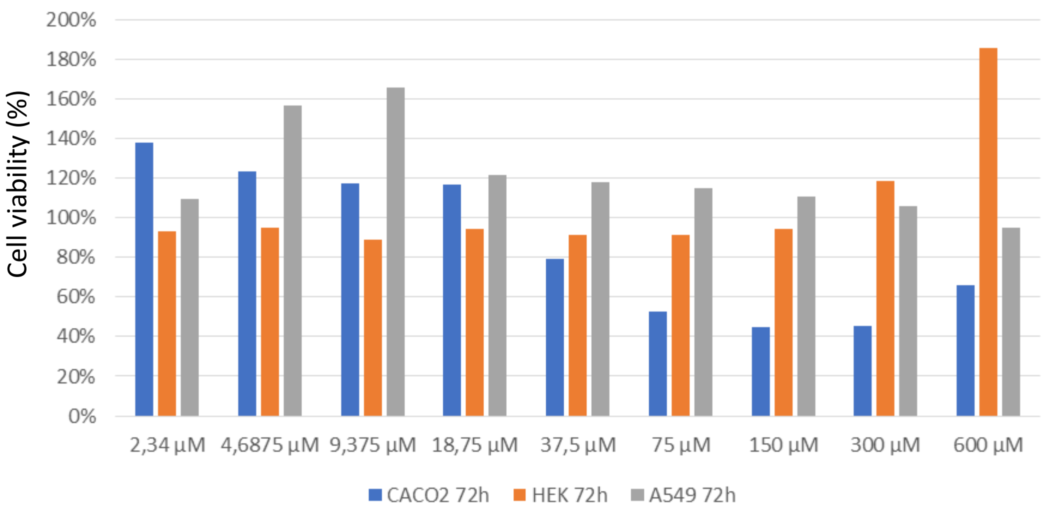

Fig. S21. Toxicity results for LQOF-G32. Bar graph showing the average percentage value of cell viability vs. concentration of LQOF-G32. Each measurement was performed in triplicate and the average value is reported.
